# Hungry scale worms: Phylogenetics of *Peinaleopolynoe* (Polynoidae, Annelida), with four new species

**DOI:** 10.3897/zookeys.932.48532

**Published:** 2020-05-12

**Authors:** Avery S. Hatch, Haebin Liew, ﻿Stéphane Hourdez, Greg W. Rouse

**Affiliations:** 1 Scripps Institution of Oceanography, University of California San Diego, La Jolla, CA 92093-0202, USA University of California San Diego La Jolla United States of America; 2 Observatoire Océanologique de Banyuls-sur-Mer, UMR 8222 CNRS-Sorbonne Université, 1 avenue Pierre Fabre, 66650 Banyuls-sur-Mer, France CNRS-Sorbonne Université Banyuls-sur-Mer France

**Keywords:** deep sea, molecular phylogeny, seeps, systematics, vents, whalefalls

## Abstract

Polynoidae Kinberg, 1856 has five branchiate genera: *Branchipolynoe* Pettibone, 1984, *Branchinotogluma* Pettibone, 1985, *Branchiplicatus* Pettibone, 1985, *Peinaleopolynoe* Desbruyères & Laubier, 1988, and *Thermopolynoe* Miura, 1994, all native to deep-sea, chemosynthetic-based habitats. Of these, *Peinaleopolynoe* has two accepted species; *Peinaleopolynoe sillardi* Desbruyères & Laubier, 1988 (Atlantic Ocean) and *Peinaleopolynoe santacatalina* Pettibone, 1993 (East Pacific Ocean). The goal of this study was to assess the phylogenetic position of *Peinaleopolynoe*, utilizing DNA sequences from a broad sampling of deep-sea polynoids. Representatives from all five branchiate genera were included, several species of which were sampled from near the type localities; *Branchinotogluma sandersi* Pettibone, 1985 from the Galápagos Rift (E/V “Nautilus”); *Peinaleopolynoe sillardi* from organic remains in the Atlantic Ocean; *Peinaleopolynoe santacatalina* from a whalefall off southern California (R/V “Western Flyer”) and *Thermopolynoe branchiata* Miura, 1994 from Lau Back-Arc Basin in the western Pacific (R/V “Melville”). Phylogenetic analyses were conducted using mitochondrial (COI, 16S rRNA, and CytB) and nuclear (18S rRNA, 28S rRNA, and H3) genes. The analyses revealed four new *Peinaleopolynoe* species from the Pacific Ocean that are formally described here: *Peinaleopolynoe orphanae* Hatch & Rouse, **sp. nov.**, type locality Pescadero Basin in the Gulf of California, Mexico (R/V “Western Flyer”); *Peinaleopolynoe elvisi* Hatch & Rouse, **sp. nov.** and *Peinaleopolynoe goffrediae* Hatch & Rouse, **sp. nov.**, both with a type locality in Monterey Canyon off California (R/V “Western Flyer”) and *Peinaleopolynoe mineoi* Hatch & Rouse, **sp. nov.** from Costa Rica methane seeps (R/V “Falkor”). In addition to DNA sequence data, the monophyly of *Peinaleopolynoe* is supported by the presence of ventral papillae on segments 12–15. The results also demonstrated the paraphyly of *Branchinotogluma* and *Lepidonotopodium* Pettibone, 1983 and taxonomic revision of these genera is required. We apply the subfamily name Lepidonotopodinae Pettibone 1983, for the clade comprised of *Branchipolynoe*, *Branchinotogluma*, *Bathykurila*, *Branchiplicatus*, *Lepidonotopodium*, *Levensteiniella* Pettibone, 1985, *Thermopolynoe*, and *Peinaleopolynoe*.

## Introduction

Within Polynoidae Kinberg, 1856, there are five genera and 25 accepted species distinguished by their presence of parapodial branchiae (Read and Fauchald 2019). Known mostly from the Pacific Ocean, branchiate polynoids are native to deep-sea, chemosynthetic-based habitats; including volcanic seamounts (Miura and Hashimoto 1991), hydrothermal vents (Pettibone 1985a), methane seeps (Chevaldonné et al. 1998), and organic remains such as whalefalls (Pettibone 1993). *Branchipolynoe* Pettibone, 1984 (nine accepted species) was initially erected for *Branchipolynoe symmytilida* Pettibone, 1984, described from the Galápagos hydrothermal vents in the eastern Pacific Ocean. *Branchinotogluma* Pettibone, 1985 (12 accepted species) was erected for *Branchinotogluma hessleri* Pettibone, 1985 and *Branchinotogluma sandersi* Pettibone, 1985, from hydrothermal vents along the East Pacific Rise, as was *Branchiplicatus* Pettibone, 1985, established for *Branchiplicatus cupreus* Pettibone, 1985. The monotypic genus *Thermopolynoe* Miura, 1994 was established for *Thermopolynoe branchiata* Miura, 1994, for specimens collected from the Lau (1750 m) and North Fiji (2000 m) basins.

The branchiate genus of focus in this study, *Peinaleopolynoe* Desbruyères & Laubier, 1988 (two currently accepted species, distinguished by their presence of four pairs of ventral papillae on segments 12–15), was erected for *Peinaleopolynoe sillardi* Desbruyères & Laubier, 1988, collected on an artificial organic fall off the coast of Spain in the northeast Atlantic. The first part of the genus name is from the Greek πειναλεoσ (peinaleos), meaning hungry or famished, and is a reference by Desbruyères and Laubier to the attraction of these worms to food falls. This was prescient, since *Peinaleopolynoe santacatalina* Pettibone, 1993 was then described for specimens associated with a whalefall, in the north east Pacific off California at 1240 m.

The most recent molecular phylogeny of Aphroditiformia (Zhang et al. 2018b) had data for five branchiate species, representing two of the five total branchiate genera. The two branchiate genera, *Branchipolynoe* (including *Branchipolynoe pettiboneae* Miura & Hashimoto, 1991, *Branchipolynoe longqiensis* Zhou, Zhang, Lu & Wang, 2017, and *B. symmytilida*) and *Branchinotogluma* (including *Branchinotogluma japonicus* (Miura & Hashimoto, 1991) and *B. sandersi*), formed a well-supported clade among the deep-sea polynoids. However, *B. sandersi* and *B. japonicus* formed a paraphyletic grade with respect to a *Branchipolynoe* clade (Zhang et al. 2018b). DNA data was previously published for *Peinaleopolynoe* sp. nov. 1 in Goffredi et al. (2017), which will be described herein as *Peinaleopolynoe orphanae* sp. nov.; and for *Peinaleopolynoe* sp. in Copley et al. (2016), which was subsequently described as *Branchinotogluma bipapillata* Zhou, Wang, Zhang & Wang, 2018. Due to the previous lack of DNA data from the type species *Peinaleopolynoe sillardi*, the phylogenetic position of *Peinaleopolynoe* was not examined until this study.

We present new DNA sequence data for a series of known and new branchiate scale worm specimens including some from nearby the type localities for *P. sillardi*, *P. santacatalina*, *B. sandersi*, and *T. branchiata*. We sequenced DNA for the following loci: mitochondrial cytochrome c oxidase subunit I (COI), 16S rRNA (16S), and cytochrome b (CytB), as well as nuclear 18S rRNA (18S), 28S rRNA (28S), and histone h3 (H3). We reassess branchiate scale worm phylogeny and the phylogenetic placement of *Peinaleopolynoe* by including representatives from all five branchiate genera. Additionally, we construct the first molecular phylogeny of *Peinaleopolynoe* by including DNA sequence data from both previously accepted *Peinaleopolynoe* spp. and describe four new *Peinaleopolynoe* spp. The morphology supporting the monophyly of the genus is examined and paraphyly of *Branchinotogluma* and *Lepidonotopodium* Pettibone, 1983 is explored.

## Materials and methods

### Sample collection and morphology

Most new samples represent a range of polynoids collected on cruises using ROVs or the HOV “Alvin” in the eastern Pacific from 2004–2019. The majority of samples were collected via Monterey Bay Aquarium Research Institute’s R/V “Western Flyer” and ROVs “Tiburon” and “Doc Ricketts”. Other eastern Pacific samples were obtained with the R/V “Falkor” and ROV “SuBastian” and the R/V “Atlantis” and HOV “Alvin”. Specimens of *B. sandersi* were collected by the E/V “Nautilus” and ROV “Hercules” from vents near the type locality for this species (Galápagos Rift vents). A specimen of *T. branchiata* was collected by the R/V “Melville” and ROV “Jason II” from a Lau Back Arc Basin hydrothermal vent, a few hundred kilometers from the type locality of vents in the North Fiji Basin in the western Pacific. A specimen of *P. sillardi* was collected from the central Atlantic at 3900 m and identified by the third author (SH). Tables 1, 2 provide further details of collection localities, deposition of types and vouchers, and GenBank accession numbers. The holotypes, most paratypes, and vouchers are deposited at the Scripps Institution of Oceanography, Benthic Invertebrate Collection (**SIO-BIC**), La Jolla, California, USA. Some paratypes are also deposited at the Museo de Zoología (Universidad de Costa Rica), San José, Costa Rica (**MZUCR**) and the Instituto de Ciencias del Mar y Limnologia, Universidad Nacional Autónoma de México (**UNAM-ICML-EMU**), Mazatlán, Mexico. The voucher for *P. sillardi* is deposited at the Muséum national d’Histoire naturelle (**MNHN-IA**), Paris, France and those of *B. sandersi* are at Harvard University’s Museum of Comparative Zoology (MCZ), Cambridge, Massachusetts, USA.

**Table 1.** Collection data, vouchers, and GenBank accession numbers (COI, 16S, 18S, 28S, H3, CytB) for all DNA sequences used in this study. New sequences are set in bold.

Species	Specimen Voucher or Source	COI	16S	18S	28S	H3	CytB	Location	Site
*Austropolaria magnicirrata* Neal, Barnich, Wiklund & Glover, 2012	Neal et al. (2012)	–	JX863896	JX863895	–	–	–	Antarctica, Amundsen Sea	Pine Island Bay
*Bathykurila guaymasensis* Pettibone, 1989	SIO–BIC A10920	–	**MN428326**	–	–	–	–	USA, California	Whalefall (Rosebud) off San Diego
	Glover et al. (2005)	DQ074766	–	DQ074765	–	–	–	USA, California	Santa Cruz Basin
*Branchinotogluma bipapillata* Zhou, Wang, Zhang & Wang, 2018	Copley et al. (2016)	KY211996	–	–	–	–	–	Indian Ocean	Southwest Indian Ridge
Branchinotogluma cf. sandersi	Norlinder et al. (2012)	JN852923	JN852889	JN852821	JN852851	–	–	Juan de Fuca	–
*Branchinotogluma elytropapillata* Zhang, Chen & Qiu, 2018	Zhang et al. (2018a)	MG799387	MG799377	MG799378	MG799380	–	–	East China Sea	Okinawa Trough, Sakai Vent Field
*Branchinotogluma hessleri* Pettibone, 1985	SIO–BIC A6316, Goffredi et al. (2017)	KY684713	**MH127414**	**MH124626**	**MH124616**	**MH120843**	**MH115419**	Mexico, Gulf of California	Alarcon Rise
*Branchinotogluma japonicus* (Miura & Hashimoto, 1991)	Zhang et al. (2018b)	–	KY753824	KY753841	KY753841	–	KY753824	East China Sea	Okinawa Trough, Sakai Vent Field
	Zhang et al. (2018a)	MG799390	–	–	–	–	–	East China Sea	Okinawa Trough, Noho Site
*Branchinotogluma ovata* Wu, Zhan & Xu, 2019	Wu et al. (2019)	MK357896	MK211416	MK211411	MK211413	–	–	West Pacific Ocean	Manus Back-Arc Basin
*Branchinotogluma pettiboneae* Wu, Zhan & Xu, 2019	Wu et al. (2019)	MK357901	MK211417	–	MK211414	–	–	West Pacific Ocean	Manus Back-Arc Basin
*Branchinotogluma sandersi* Pettibone, 1985	SIO–BIC A6321, Goffredi et al. (2017)	KY684716	**MH127416**	**MH124627**	**MH124617**	**MH120844**	**MH115420**	Mexico, Gulf of California	Alarcon Rise
	MCZ70173.1	**MH115399**	–	–	–	–	–	Ecuador	Galápagos Rift, Tempus Fugit Vent Field
	MCZ70173.2	**MH115400**	–	–	–	–	–	Ecuador	Galápagos Rift, Tempus Fugit Vent Field
	MCZ70173.3	**MH115401**	–	–	–	–	–	Ecuador	Galápagos Rift, Tempus Fugit Vent Field
	MCZ70173.4	**MH115402**	–	–	–	–	–	Ecuador	Galápagos Rift, Tempus Fugit Vent Field
	MCZ70173.5	**MH115403**	–	–	–	–	–	Ecuador	Galápagos Rift, Tempus Fugit Vent Field
	MCZ70173.6	**MH115404**	–	–	–	–	–	Ecuador	Galápagos Rift, Tempus Fugit Vent Field
	MCZ70173.7	**MH115405**	–	–	–	–	–	Ecuador	Galápagos Rift, Tempus Fugit Vent Field
*Branchinotogluma segonzaci* (Miura & Desbruyères, 1995)	Wu et al. (2019)	MK357906	MK211418	MK211412	–	–	–	West Pacific Ocean	Manus Back-Arc Basin
*Branchinotogluma* sp. nov. 1	SIO–BIC A6152, Goffredi et al. (2017)	KY684721	**MH127420**	**MH124625**	**MH124615**	–	**MH115418**	Mexico, Gulf of California	Alarcon Rise
*Branchinotogluma* sp. nov. 2	SIO–BIC A6156, Goffredi et al. (2017)	KY684723	**MH127419**	**MH124624**	**MH124614**	–	**MH115417**	Mexico, Gulf of California	Alarcon Rise
*Branchinotogluma* sp. nov. 3	SIO–BIC A6331, Goffredi et al. (2017)	KY684725	**MH127415**	**MH124623**	**MH124613**	**MH120842**	**MH115416**	Mexico, Gulf of California	Pescadero Basin
*Branchinotogluma* sp. nov. 4	SIO–BIC A6157, Goffredi et al. (2017)	KY684728	**MH127417**	**MH124622**	**MH124620**	–	**MH115415**	Mexico, Gulf of California	Pescadero Basin
*Branchinotogluma trifurcus* (Miura & Desbruyères, 1995)	Wu et al. (2019)	MK357905	MK211415	MK211410	–	–	–	West Pacific Ocean	Manus Back-Arc Basin
*Branchiplicatus cupreus* Pettibone, 1985	SIO–BIC A6160, Goffredi et al. (2017)	KY684706	**MH127418**	**MH124628**	–	**MH120845**	**MH115421**	Mexico, Gulf of California	Pescadero Basin
*Branchipolynoe eliseae* Lindgren, Hatch, Hourdez, Seid & Rouse, 2019	SIO–BIC A6548, Lindgren et al. (2019)	MH369878	MH396826	–	–	–	–	Costa Rica	Jaco Scar
*Branchipolynoe halliseyae* Lindgren, Hatch, Hourdez, Seid & Rouse, 2019	SIO–BIC A6532, Lindgren et al. (2019)	MH369858	MH396795	–	–	–	–	Costa Rica	Mound 12
*Branchipolynoe kajsae* Lindgren, Hatch, Hourdez, Seid & Rouse, 2019	SIO–BIC A2161, Lindgren et al. (2019)	MH369859	MH396800	–	–	–	–	Costa Rica	Jaco Scar
*Branchipolynoe longqiensis* Zhou, Zhang, Lu & Wang, 2017	Zhang et al. (2018b)	KY753826	KY753826	KY753847	KY753847	–	KY753826	Indian Ocean	Southwest Indian Ridge, Dragon Vent Field
*Branchipolynoe meridae* Lindgren, Hatch, Hourdez, Seid & Rouse, 2019	SIO–BIC A2131, Lindgren et al. (2019)	MH369884	MH396829	–	–	–	–	Costa Rica	Jaco Scar
*Branchipolynoe pettiboneae* Miura & Hashimoto, 1991	Zhang et al. (2018a)	MG799393	–	–	–	–	–	East China Sea	Okinawa Trough
	Zhang et al. (2018b)	–	KY753825	KY753840	KY753840	–	KY753825	South China Sea	Seep
*Branchipolynoe seepensis* Pettibone, 1986	SIO–BIC A6553, Lindgren et al. (2019)	MH369885	MH596848	–	–	–	–	USA, Gulf of Mexico	Florida Escarpment
*Branchipolynoe symmytilida* Pettibone, 1984	Halanych et al. (2001); Hurtado et al. (2004)	AY646021	AF315055	–	–	–	–	Ecuador	Galápagos Rift
*Branchipolynoe tjiasmantoi* Lindgren, Hatch, Hourdez, Seid & Rouse, 2019	SIO–BIC A8511, Lindgren et al. (2019)	MH369947	MH396830	–	–	–	–	Lau Back-Arc Basin	Kilo Moana
*Gesiella jameensis* (Hartmann-Schröder, 1974)	Gonzalez et al. (2017a); Gonzalez et al. (2017b)	KY454429	KY454412	KY454403	KY823476	–	–	Spain, Canary Islands	Lanzarote, Túnel de la Átlantida
*Lepidonotopodium fimbriatum* Pettibone, 1983	SIO–BIC A6153, Goffredi et al. (2017)	KY684717	**MN428327**	**MN428333**	**MN428320**	–	–	Mexico, Gulf of California	Alarcon Rise
*Lepidonotopodium okinawae* (Sui & Li, 2017)	Zhang et al. (2018b)	–	KY753828	KY753842	KY753842	–	KY753828	East China Sea	Okinawa Trough, Sakai Vent Field
	Zhang et al. (2018a)	MG799384	–	–	–	–	–	East China Sea	Okinawa Trough
*Lepidonotopodium* sp. nov.	SIO–BIC A6317, Goffredi et al. (2017)	KY684715	**MN428328**	**MN428334**	**MN428321**	–	–	Mexico, Gulf of California	Alarcon Rise
*Lepidonotopodium williamsae* Pettibone, 1984	SIO–BIC A6318, Goffredi et al. (2017)	KY684714	**MN428329**	**MN428335**	**MN428322**	–	–	Mexico, Gulf of California	Alarcon Rise
*Levensteiniella iris* Hourdez & Desbruyères, 2003	Zhang et al. (2018b)	KY753827	KY753827	KY753848	KY753848	–	KY753827	East Scotia Ridge	Segment E9
*Levensteiniella undomarginata* Zhang, Chen & Qiu, 2018	Zhang et al. (2018a)	MG799385	MG799376	MG799379	MG799381	–	–	East China Sea	Okinawa Trough
*Peinaleopolynoe elvisi* sp. nov.	SIO–BIC A9699	**MN431777**	–	–	–	–	–	Costa Rica	Jaco Scar
	SIO–BIC A9871	**MN431779**	–	–	–	–	–	Costa Rica	Seamount 1
	SIO–BIC A9870	**MN431778**	–	–	–	–	–	Costa Rica	Seamount 1
	SIO–BIC A9752	**MN431780**	–	–	–	–	–	Costa Rica	Jaco Scar
	SIO–BIC A8488	**MH115413**	**MH127422**	**MH124629**	**MH124618**	**MH120847**	**MH115423**	USA, California	Monterey Canyon
*Peinaleopolynoe goffrediae* sp. nov.	SIO–BIC A5485	**MN431782**	**MN428330**	**MN428336**	**MN428323**	**MN431801**	–	USA, California	Monterey Canyon
	SIO–BIC A5464	**MN431783**	–	–	–	–	–	USA, California	Monterey Canyon
*Peinaleopolynoe mineoi* sp. nov.	SIO–BIC A10071	**MN431775**	–	–	–	–	–	Costa Rica	Mound 12
	SIO–BIC A9919	**MN431773**	**MN428331**	**MN428337**	**MN428324**	**MN431802**	**MN431803**	Costa Rica	Mound 11
	SIO–BIC A10070	**MN431774**	–	–	–	–	–	Costa Rica	Mound 12
	SIO–BIC A9709	**MN431776**	–	–	–	–	–	Costa Rica	Mound 12
*Peinaleopolynoe orphanae* sp. nov.	SIO–BIC A6154, Goffredi et al. (2017)	KY684708	–	–	–	–	–	Mexico, Gulf of California	Pescadero Basin
	SIO–BIC A6312, Goffredi et al. (2017)	KY684709	–	–	–	–	–	Mexico, Gulf of California	Pescadero Basin
	SIO–BIC A6163	**MH115406**	–	–	–	–	–	Mexico, Gulf of California	Pescadero Basin
	SIO–BIC A6166	**MH115407**	–	–	–	–	–	Mexico, Gulf of California	Pescadero Basin
	SIO–BIC A9989	**MN431785**	–	–	–	–	–	Mexico, Gulf of California	Pescadero Basin, Auka Vent Field, Z Mound
	SIO–BIC A9988	**MN431790**	–	–	–	–	–	Mexico, Gulf of California	Pescadero Basin, Auka Vent Field, Z Mound
	SIO–BIC A10021	**MN431791**	–	–	–	–	–	Mexico, Gulf of California	Pescadero Basin, Auka Vent Field, Z Mound
	SIO–BIC A10025	**MN431789**	–	–	–	–	–	Mexico, Gulf of California	Pescadero Basin, Auka Vent Field, Z Mound
	SIO–BIC A10037	**MN431792**	–	–	–	–	–	Mexico, Gulf of California	Pescadero Basin, Auka Vent Field, Z Mound
	SIO–BIC A10020	**MN431793**	–	–	–	–	–	Mexico, Gulf of California	Pescadero Basin, Auka Vent Field, Z Mound
*Peinaleopolynoe orphanae* sp. nov.	SIO–BIC A10922	**MN431794**	–	–	–	–	–	Mexico, Gulf of California	Pescadero Basin, JaichMaa Vent Field, Cavern Tay Ujaa
	SIO–BIC A6151, Goffredi et al. (2017)	KY684727	**MH127423**	**MH124630**	**MH124619**	**MH120841**	–	Mexico, Gulf of California	Pescadero Basin
	SIO–BIC A6150	**MH115409**	–	–	–	–	–	Mexico, Gulf of California	Pescadero Basin
	SIO–BIC A10926	**MN431784**	–	–	–	–	–	USA, California	Monterey Canyon, vesicomyid clam bed near whalefall (Ruby)
	SIO–BIC A10921	**MN431786**	–	–	–	–	–	Mexico, Gulf of California	Pescadero Basin, JaichMaa Vent Field, Cavern Tay Ujaa
	SIO–BIC A10026	**MN431795**	–	–	–	–	–	Mexico, Gulf of California	Pescadero Basin, Auka Vent Field, Z Mound
	SIO–BIC A10022	**MN431796**	–	–	–	–	–	Mexico, Gulf of California	Pescadero Basin, Auka Vent Field, Z Mound
	SIO–BIC A10001	**MN431797**	–	–	–	–	–	Mexico, Gulf of California	Pescadero Basin, JaichMaa Vent Field, Cavern Tay Ujaa
	SIO–BIC A10023	**MN431799**	–	–	–	–	–	Mexico, Gulf of California	Pescadero Basin, Auka Vent Field, Z Mound
	SIO–BIC A6155	**MH115408**	–	–	–	–	–	Mexico, Gulf of California	Pescadero Basin
	SIO–BIC A9996	**MN431800**	–	–	–	–	–	Mexico, Gulf of California	Pescadero Basin, JaichMaa Vent Field, Cavern Tay Ujaa
	SIO–BIC A10923	**MN431798**	–	–	–	–	–	Mexico, Gulf of California	Pescadero Basin, JaichMaa Vent Field, Cavern Tay Ujaa
	SIO–BIC A10003	**MN431787**	–	–	–	–	–	Mexico, Gulf of California	Pescadero Basin, JaichMaa Vent Field, Cavern Tay Ujaa
	SIO–BIC A10036	**MN431788**	–	–	–	–	–	Mexico, Gulf of California	Pescadero Basin, Auka Vent Field, Z Mound
*Peinaleopolynoe santacatalina* Pettibone, 1993	SIO–BIC A8490	**MH115412**	–	–	–	–	–	USA, California	Del Mar Seeps
	SIO–BIC A10924	**MN431781**	–	–	–	–	–	USA, California	Whalefall (Rosebud) off San Diego
	SIO–BIC A8489	**MH115410**	–	–	–	–	–	USA, California	Whalefall (Rosebud) off San Diego
	SIO–BIC A8487	**MH115411**	**MH127413**	**MH124621**	**MH124612**	**MH120846**	**MH115422**	USA, California	Whalefall (Rosebud) off San Diego
*Peinaleopolynoe sillardi* Desbruyères & Laubier, 1988	MNHN–IA–2010–399	**MH115414**	**MH127421**	–	–	–	–	South Atlantic Ocean	–
Pelagomacellicephala cf. iliffei	Gonzalez et al. (2017a); Gonzalez et al. (2017b)	KY454435	KY454420	KY454408	KY823474	–	–	Bahamas, Eleuthera	Preachers Blue Hole
*Thermopolynoe branchiata* Miura, 1994	SIO–BIC A10925	**MN431772**	**MN428332**	**MN428338**	**MN428325**	–	–	Lau Back-Arc Basin	Kilo Moana

Prior to preservation, whole specimens were generally relaxed with 7% MgCl_2_ in fresh water and photographed alive using Leica MZ8 or MZ9.5 stereomicroscopes with a Canon EOS Rebel T6i attachment. They were then fixed in either 95% ethanol for DNA extraction or 10% formaldehyde in seawater for morphological work. For those fixed in formalin, some elytra were also fixed in 95% ethanol. After a day, specimens preserved in formalin were rinsed and transferred to 50% ethanol. Post-preservation, specimens of the new species *Peinaleopolynoe orphanae* sp. nov., *Peinaleopolynoe elvisi* sp. nov., *Peinaleopolynoe goffrediae* sp. nov., and *Peinaleopolynoe mineoi* sp. nov. were examined (Table 2) and photographed using the Leica S8 APO, DMR HC, and/or Leica MZ9.5 microscopes with a Canon EOS Rebel T6i attachment.

**Table 2.** Detailed collection data and vouchers for *Peinaleopolynoe* specimens examined in the descriptions.

Species	Voucher	Location	Site	Latitude / Longitude	Depth	Collection Date	Type Status
*Peinaleopolynoe santacatalina*	SIO-BIC A8490	USA, California	Del Mar Seeps	32°54.25'N, 117°46.94'W	1020–1036 m	19.05.13	Specimen
	SIO-BIC A10924	USA, California	Whalefall (Rosebud) off San Diego	32°46.30'N, 117°27.18'W	850–858 m	20.06.14	Specimen
	SIO-BIC A8489	USA, California	Whalefall (Rosebud) off San Diego	32°46.30'N, 117°27.18'W	850–858 m	20.06.14	Specimen
	SIO-BIC A8487	USA, California	Whalefall (Rosebud) off San Diego	32°46.62'N, 117°29.26'W	842–857 m	18.05.13	Specimen
	SIO-BIC A10927	USA, California	Whalefall (Rosebud) off San Diego	32°46.62'N, 117°29.26'W	842–857 m	18.05.13	Specimen
	SIO-BIC A8565	USA, California	Whalefall (Rosebud) off San Diego	32°46.62'N, 117°29.26'W	842–857 m	18.05.13	Specimen
	SIO-BIC A8566	USA, California	Whalefall (Rosebud) off San Diego	32°46.62'N, 117°29.26'W	842–857 m	18.05.13	Specimen
	SIO-BIC A8567	USA, California	Whalefall (Rosebud) off San Diego	32°46.62'N, 117°29.26'W	842–857 m	18.05.13	Specimen
*Peinaleopolynoe orphanae* sp. nov.	SIO-BIC A6151	Mexico, Gulf of California	Pescadero Basin	23°57.23'N, 108°51.73'W	3700 m	24.04.15	Holotype
	SIO-BIC A8597	Mexico, Gulf of California	Pescadero Basin	23°57.23'N, 108°51.73'W	3700 m	24.04.15	Paratype
	SIO-BIC A6154	Mexico, Gulf of California	Pescadero Basin	23°57.58'N, 108°51.78'W	3676–3756 m	18.04.15	Paratype
	SIO-BIC A6312	Mexico, Gulf of California	Pescadero Basin	24°0.00'N, 108°49.98'W	3676 m	19.04.15	Paratype
	SIO-BIC A6163	Mexico, Gulf of California	Pescadero Basin	23°57.58'N, 108°51.78'W	3676–3756 m	18.04.15	Paratype
	SIO-BIC A6166	Mexico, Gulf of California	Pescadero Basin	23°57.23'N, 108°51.73'W	3700 m	24.04.15	Paratype
	SIO-BIC A9989	Mexico, Gulf of California	Pescadero Basin, Auka Vent Field, Z Mound	23°57.37'N, 108°51.71'W	3688 m	17.11.18	Paratype
	SIO-BIC A9988	Mexico, Gulf of California	Pescadero Basin, Auka Vent Field, Z Mound	23°57.41'N, 108°51.82'W	3670 m	17.11.18	Paratype
	SIO-BIC A10021	Mexico, Gulf of California	Pescadero Basin, Auka Vent Field, Z Mound	23°57.37'N, 108°51.71'W	3687 m	21.11.18	Paratype
	SIO-BIC A10025	Mexico, Gulf of California	Pescadero Basin, Auka Vent Field, Z Mound	23°57.37'N, 108°51.71'W	3687 m	21.11.18	Paratype
	SIO-BIC A10037	Mexico, Gulf of California	Pescadero Basin, Auka Vent Field, Z Mound	23°57.37'N, 108°51.71'W	3688 m	21.11.18	Paratype
	SIO-BIC A10020	Mexico, Gulf of California	Pescadero Basin, Auka Vent Field, Z Mound	23°57.37'N, 108°51.71'W	3687 m	21.11.18	Paratype
	SIO-BIC A10922	Mexico, Gulf of California	Pescadero Basin, JaichMaa Vent Field, Cavern Tay Ujaa	23°56.51'N, 108°51.34'W	3692 m	18.11.18	Paratype
	SIO-BIC A6150	Mexico, Gulf of California	Pescadero Basin	23°57.58'N, 108°51.78'W	3676–3756 m	18.04.15	Paratype
	SIO-BIC A10926	USA, California	Monterey Canyon, vesicomyid clam bed near whalefall (Ruby)	36°46.33'N, 122°4.99'W	2900 m	28.10.10	Paratype
	SIO-BIC A10921	Mexico, Gulf of California	Pescadero Basin, JaichMaa Vent Field, Cavern Tay Ujaa	23°56.51'N, 108°51.34'W	3692 m	18.11.18	Paratype
*Peinaleopolynoe orphanae* sp. nov.	SIO-BIC A10026	Mexico, Gulf of California	Pescadero Basin, Auka Vent Field, Z Mound	23°57.37'N, 108°51.71'W	3687 m	21.11.18	Paratype
	SIO-BIC A10022	Mexico, Gulf of California	Pescadero Basin, Auka Vent Field, Z Mound	23°57.37'N, 108°51.71'W	3687 m	21.11.18	Paratype
	SIO-BIC A10001	Mexico, Gulf of California	Pescadero Basin, JaichMaa Vent Field, Cavern Tay Ujaa	23°56.49'N, 108°51.35'W	3666 m	18.11.18	Paratype
	SIO-BIC A10024	Mexico, Gulf of California	Pescadero Basin, Auka Vent Field, Z Mound	23°57.37'N, 108°51.71'W	3687 m	21.11.18	Paratype
	SIO-BIC A10023	Mexico, Gulf of California	Pescadero Basin, Auka Vent Field, Z Mound	23°57.37'N, 108°51.71'W	3687 m	21.11.18	Paratype
	SIO-BIC A6155	Mexico, Gulf of California	Pescadero Basin	23°57.58'N, 108°51.78'W	3676–3756 m	18.04.15	Paratype
	SIO-BIC A9996	Mexico, Gulf of California	Pescadero Basin, JaichMaa Vent Field, Cavern Tay Ujaa	23°56.48'N, 108°51.35'W	3667 m	18.11.18	Paratype
	SIO-BIC A10923	Mexico, Gulf of California	Pescadero Basin, JaichMaa Vent Field, Cavern Tay Ujaa	23°56.51'N, 108°51.34'W	3692 m	18.11.18	Paratype
	SIO-BIC A10003	Mexico, Gulf of California	Pescadero Basin, JaichMaa Vent Field, Cavern Tay Ujaa	23°56.51'N, 108°51.34'W	3692 m	18.11.18	Paratype
	SIO-BIC A10036	Mexico, Gulf of California	Pescadero Basin, Auka Vent Field, Z Mound	23°57.37'N, 108°51.71'W	3688 m	21.11.18	Paratype
*Peinaleopolynoe elvisi* sp. nov.	SIO-BIC A8488	USA, California	Monterey Canyon, Whalefall (Patrick)	36°46.33'N, 122°4.99'W	1820 m	20.11.09	Holotype
	SIO-BIC A9699	Costa Rica	Jaco Scar	9°6.88'N, 84°50.14'W	1845 m	18.10.18	Paratype
	SIO-BIC A9871	Costa Rica	Seamount 1	8°52.60'N, 85°7.34'W	2091 m	29.10.18	Paratype
	SIO-BIC A9870	Costa Rica	Seamount 1	8°52.60'N, 85°7.34'W	2091 m	29.10.18	Paratype
	SIO-BIC A9752	Costa Rica	Jaco Scar	9°6.91'N, 84°50.39'W	1887 m	22.10.18	Paratype
*Peinaleopolynoe goffrediae* sp. nov.	SIO-BIC A5485	USA, California	Monterey Canyon	36°36.79'N, 122°26.01'W	2891 m	29.09.04	Holotype
	SIO-BIC A5464	USA, California	Monterey Canyon	36°36.79'N, 122°26.01'W	2891 m	29.09.04	Paratype
*Peinaleopolynoe mineoi* sp. nov.	SIO-BIC A10071	Costa Rica	Mound 12	8°55.99'N, 84°18.45'W	1011 m	8.01.19	Holotype
	SIO-BIC A9919	Costa Rica	Mound 11	8°55.33'N, 84°18.27'W	1010 m	3.11.18	Paratype
	SIO-BIC A10070	Costa Rica	Mound 12	8°55.99'N, 84°18.45'W	1011 m	8.01.19	Paratype
	SIO-BIC A9709	Costa Rica	Mound 12	8°55.80'N, 84°18.70'W	992 m	20.10.18	Paratype

### DNA extraction, amplification, and sequencing

DNA from samples fixed and preserved in 95% ethanol was extracted using the Zymo Research DNA-Tissue Miniprep kit, following the manufacturer’s protocol. Partial mitochondrial cytochrome c oxidase subunit I (COI) DNA sequences were obtained for these specimens for ‘species’ delimitation (Table 1). Representatives from each ‘species’ within the combined COI data set from this study and terminals that had only been sequenced for COI in Goffredi et al. (2017) were then sequenced for mitochondrial (16S rRNA (16S) and cytochrome b (CytB)) and nuclear (18S rRNA (18S), 28S rRNA (28S), and histone h3 (H3)) genes. All sequences obtained are deposited in GenBank (Table 1). Amplification was carried out using a PCR mixture of 12.5µl Apex 2.0× Taq Red DNA Polymerase Master Mix (Genesee Scientific), 1µl each of the appropriate forward and reverse primers (10µM), 8.5µl of ddH_2_O, and 2µl of eluted DNA, or when amplification using this mixture failed, 12.5µl Apex 2.0× Taq Red DNA Polymerase Master Mix (Genesee Scientific) was substituted with 12.5µl Conquest PCR 2.0× Master Mix 1 (Lamda Biotech). DNA sequencing was completed with the following PCR primers (Table 3) and temperature profiles, performed in a thermal cycler (Eppendorf). Final PCR products were purified with ExoSAP-IT (USB Affimetrix, Ohio, USA), and Sanger sequencing was performed by Eurofins Genomics (Louisville, KY) or Retrogen, Inc. (San Diego, CA).

**Table 3.** List of primers used for amplification and sequencing, with original references.

Gene	Primer name & direction	Primer sequence (5’-3’ direction)	Source
COI	LCO1490 (F)	GGTCAACAAATCATAAAGATATTGG	Folmer et al. (1994)
COI	HCO2198 (R)	TAAACTTCAGGGTGACCAAAAAATCA	Folmer et al. (1994)
16S	16SarL (F)	CGCCTGTTTATCAAAAACAT	Palumbi (1996)
16S	16SbrH (R)	CCGGTCTGAACTCAGATCACGT	Palumbi (1996)
18S	18S-1F (F)	TACCTGGTTGATCCTGCCAGTAG	Giribet et al. (1996)
18S	18S-5R (R)	CTTGGCAAATGCTTTCGC	Giribet et al. (1996)
18S	18S-3F (F)	GTTCGATTCCGGAGAGGGA	Giribet et al. (1996)
18S	18S-bi (R)	GAGTCTCGTTCGTTATCGGA	Whiting et al. (1997)
18S	18S-a2.0 (F)	ATGGTTGCAAAGCTGAAAC	Whiting et al. (1997)
18S	18S-9R (R)	GATCCTTCCGCAGGTTCACCTAC	Giribet et al. (1996)
28S	Po28F1 (F)	TAAGCGGAGGAAAAGAAAC	Struck et al. (2006)
28S	Po28R4 (R)	GTTCACCATCTTTCGGGTCCCAAC	Struck et al. (2006)
H3	H3F (F)	ATGGCTCGTACCAAGCAGAC(ACG)GC	Colgan et al. (1998)
H3	H3R (R)	ATATCCTT(AG)GGCAT(AG)AT(AG)GTGAC	Colgan et al. (1998)
CytB	CytB-52F (F)	TCCCTTATTGATCTTCCTGCC	This study
CytB	CytB-649R (R)	CAGAGTTTGAGTTTAGTCCTAAAGG	This study
CytB	CytB-62F (F)	ACCTTCCTGCCCCTAGTAAT	This study
CytB	CytB-664R (R)	GGAAGGGGATTTTATCTGAGTTTG	This study
CytB	CytB-487F (F)	GAATGATTATGAGGAGGATTTGCC	This study
CytB	CytB-1077R (R)	GTAAAGAAGGGTAAAGATTTGGCC	This study

Up to 690 bp of COI were amplified with the reaction protocol LCO1490/HCO2198: 94 °C/180 s – (94 °C/30 s – 47 °C/45 s – 72 °C/60 s) * 5 cycles – (94 °C/30 s – 52 °C/45 s – 72 °C/60 s) * 30 cycles – 72 °C/300 s. Up to 506 bp of 16S were amplified with the reaction protocol 16SarL/16SbrH: 95 °C/180 s – (95 °C/40 s – 50 °C/40 s – 72 °C/50 s) * 35 cycles – 72 °C/300 s. Up to 1870 bp of 18S were amplified with the following reaction protocols. 1F/5R and a2.0/9R: 95 °C/180 s – (95 °C/30 s – 50 °C/30 s – 72 °C/90 s) * 40 cycles – 72 °C/480 s. 3F/bi: 95 °C/180 s – (95 °C/30 s – 52 °C/30 s – 72 °C/90 s) * 40 cycles – 72 °C/480 s. Up to 1147 bp of 28S were amplified with the reaction protocol Po28F1/Po28R4: 95 °C/180 s – (95 °C/30 s – 55 °C/40 s – 72 °C/75 s) * 40 cycles – 72 °C/300 s. Up to 1002 bp of CytB were amplified with the reaction protocol CytB-52F/CytB-649R, CytB-62F/CytB-664R and CytB-487F/CytB-1077R: 94 °C/240 s – (94 °C/60 s – 48 °C/60 s – 72 °C/120 s) * 40 cycles – 72 °C/360 s. Up to 334 bp of H3 were amplified with the reaction protocol H3F/H3R: 95 °C/180 s – (95 °C/30 s – 53 °C/45 s – 72 °C/45 s) * 40 cycles – 72 °C/300 s.

### Phylogenetic analyses

Consensus sequences were created via De Novo Assembly on Geneious v.11.0.5 (Kearse et al. 2012) with default settings. Alignments of the newly generated sequences, along with data for the six genes logged on GenBank from several different studies (Table 1), were performed for each gene using MAFFT v.7 server (Katoh and Standley 2013) with the G-INS-1 progressive method. In this study, we referred to the *Branchinotogluma sandersi* sequences JN852923, JN852889, JN852821, and JN852851, sourced from Norlinder et al. (2012), as Branchinotogluma cf. sandersi, because the specimen was collected from Juan de Fuca hydrothermal vents in the northeast Pacific, as opposed to from the type locality along the Galápagos Rift. We have included *B. sandersi* sequences from the type locality and the Gulf of California (Table 1). Aligned sequences for COI, 16S, 18S, 28S, H3, and CytB were concatenated using SequenceMatrix v.1.8 (Vaidya et al. 2011). A maximum likelihood (ML) analysis was performed using RAxML v.8.1.22 (Stamatakis 2014) on the concatenated data set partitioned by gene, using the model GTR+G. Node support was assessed via the thorough bootstrapping option (with 1000 pseudoreplicates). The deep-sea polynoids *Austropolaria magnicirrata* Neal, Barnich, Wiklund & Glover, 2012, *Gesiella jameensis* (Hartmann-Schröder, 1974), and Pelagomacellicephala cf. iliffei were chosen as the most appropriate outgroup based on previous phylogenetic results (Gonzalez et al. 2017b). A Bayesian inference (BI) analysis of the concatenated data partitioned by gene was also conducted using Mr. Bayes v.3.2.6 (Ronquist et al. 2012). Best-fit models for these partitions were selected using the Akaike information criterion (AIC) in jModelTest 2.1.10 v.20160303 (Guindon and Gascuel 2003; Darriba et al. 2012). COI, 16S, 18S, 28S, and CytB were assigned the GTR+I+G model; H3 was assigned the HKY+G model. A maximum parsimony (MP) analysis was conducted using PAUP* v.4.0a165 (Swofford 2002), using heuristic searches with the tree-bisection-reconnection branch-swapping algorithm and 100 random addition replicates. Support values were determined using 100 jackknife replicates each with 100 random addition searches and heuristic search with tree-bisection-reconnection. The ML tree of the combined analysis of COI, 16S, 18S, 28S, H3, and CytB was annotated with ML bootstrap percentages from RAxML, BI posterior probability, and MP jackknife support values. Minimum uncorrected interspecific pairwise distances and maximum uncorrected intraspecific distances were calculated for the *Peinaleopolynoe*COI dataset with PAUP* v.4.0a165 (Swofford 2002).

### Haplotype networks

Median-joining haplotype networks (Bandelt et al. 1999) with geographic locality coding were generated for the COI data obtained for *P. santacatalina* (trimmed to 517 bp), *P. orphanae* sp. nov. (594 bp), *P. elvisi* sp. nov. (587 bp), and *P. mineoi* sp. nov. (678 bp) using PopART v.1.7 (Leigh and Bryant 2015). The haplotype network produced for *P. orphanae* sp. nov. was also coded for elytral color.

### Character transformations

A cutdown ML molecular phylogeny of *Peinaleopolynoe* with its sister group, a clade composed of *Branchinotogluma* sp. nov. 1 and *B. bipapillata*, was generated using the same data (realigned) and with the same parameters with RAxML. Character transformations for two morphological features were then mapped onto this tree using Mesquite v.3.6 (Maddison and Maddison 2018). The Mk1 likelihood model was used for the transformations, because this incorporates branch length information into the transformation. The morphological characters and states used were:

1 Ventral segmental papillae and/or lamellae: State 0, Males with two pairs of papillae on segments 12–13 and four pairs of lamellae on segments 14–17, and females with five pairs of papillae on segments 11–15; State 1, Four pairs of papillae on segments 12–15.

2 Elytral number: State 0, 10 pairs of elytra; State 1, 9 pairs of elytra.

## Results

### Phylogeny and species delimitation

**Figure 1.**
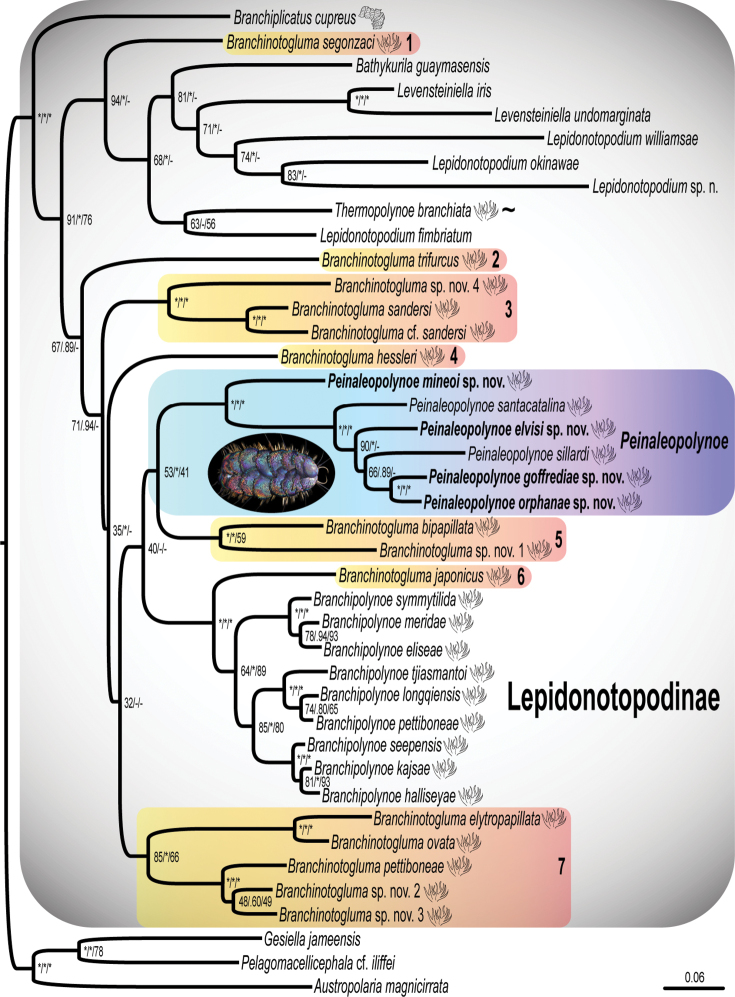
Maximum likelihood (ML) tree of the combined analysis from six genes (COI, 16S, 18S, 28S, H3, CytB) aligned with MAFFT and then concatenated. Numbers next to nodes are ML bootstrap percentages from RAxML, Bayesian inference (BI) posterior probability, and maximum parsimony (MP) jackknife support values, separated by slashes. Key: * indicates 95% bootstrap/jackknife or greater and 0.95 posterior probability or greater. – indicates the node was not found. Branchiae drawings at terminals indicate presence of arborescent or plicate branchiae; ~ indicates that on segments with two groups of branchiae, the position in *T. branchiata* is split into anterior and posterior groups, as opposed to upper and lower groups in remaining taxa. The seven paraphyletic groups of *Branchinotogluma* are highlighted in a yellow-orange gradient.

All analyses (Fig. 1) recovered *B. cupreus* as the sister taxon to a well-supported clade of the remaining ingroup taxa, a clade that comprised the remaining four branchiate genera, as well as the non-branchiate scale worms *Levensteiniella* spp., *Lepidonotopodium* spp., and *Bathykurila guaymasensis* Pettibone, 1989. In the ML and MP analyses, the branchiate *T. branchiata* was recovered as the sister taxon to *Lepidonotopodium fimbriatum* Pettibone, 1983 and nested among other non-branchiates: *Lepidonotopodium* spp., *Levensteiniella* spp. and *B. guaymasensis*. *Lepidonotopodium*, with *L. fimbriatum* as the type, was found to be paraphyletic (Fig. 1).

The ML, BI, and MP analyses (Fig. 1) of the concatenated data set were not congruent at deeper nodes and this is reflected in the low support for some of these nodes. However, all analyses showed the same topology for relationships within *Branchinotogluma* at shallower nodes (3, 4, 5, 6 and 7 discussed below). The BI placement of *Branchinotogluma segonzaci* (Miura & Desbruyères, 1995) and *Branchinotogluma trifurcus* (Miura & Desbruyères, 1995) (Suppl. material 1: Fig. S1) was congruent with the ML analysis (1 and 2 discussed below), but these taxa had unresolved placement (collapsed nodes) in the MP analysis (Suppl. material 1: Fig. S2). Although differing at some nodes with regards to the clade composed of *Levensteiniella* spp., *Lepidonotopodium* spp., *B. guaymasensis*, and *T. branchiata*, both the BI (Suppl. material 1: Fig. S1) and the MP (Suppl. material 1: Fig. S2) analyses recovered *Lepidonotopodium* as non-monophyletic. The ML, BI, and MP analyses all supported the monophyly of *Branchipolynoe* and recovered the same relationships among the nine *Branchipolynoe* spp., which were the same as reported in Lindgren et al. (2019).

The ML, BI, and MP analyses also recovered *Branchinotogluma* as non-monophyletic, with the genus scattered across the ingroup (Fig. 1, see numbers 1–7):

1 *Branchinotogluma segonzaci* formed a well-supported clade with, and was the sister taxon to, *Levensteiniella* spp., *Lepidonotopodium* spp., *B. guaymasensis*, and *T. branchiata*.

2 *Branchinotogluma trifurcus* was recovered as sister taxon (with low support) to the remaining *Branchinotogluma* spp. analyzed in this study (excluding *B. segonzaci*), *Peinaleopolynoe*, and *Branchipolynoe*.

3 *Branchinotogluma* sp. nov. 4, *B. sandersi*, and Branchinotogluma cf. sandersi formed a well-supported clade; *B. sandersi* was the sister taxon to Branchinotogluma cf. sandersi, which together formed the sister group to *B.* sp. nov. 4.

4 *Branchinotogluma hessleri* (the type species of *Branchinotogluma*) was recovered as sister taxon (with low support) to the remaining *Branchinotogluma* spp. analyzed in this study (excluding *B. segonzaci*, *B. trifurcus*, and the aforementioned clade 3), *Peinaleopolynoe*, and *Branchipolynoe*.

5 *Branchinotogluma bipapillata* and *B.* sp. nov. 1 formed a well-supported clade that was the sister group to *Peinaleopolynoe*, though there was low support for this relationship in the MP analysis (Fig. 1).

6 *Branchinotogluma japonicus* was recovered as the sister taxon (with high support) to *Branchipolynoe* in all three analyses.

7 The last *Branchinotogluma* clade was composed of two smaller clades: *Branchinotogluma elytropapillata* Zhang, Chen & Qiu, 2018 and *Branchinotogluma ovata* Wu, Zhan & Xu, 2019 formed a well-supported clade, which was the sister group (low MP support) to a clade composed of *Branchinotogluma pettiboneae* Wu, Zhan & Xu, 2019, *Branchinotogluma* sp. nov. 2, and *Branchinotogluma* sp. nov. 3.

The focus in this study, *Peinaleopolynoe*, was a well-supported clade in all analyses (Fig. 1) and the relationships within *Peinaleopolynoe* were congruent in the ML and BI analyses. *Peinaleopolynoe mineoi* sp. nov., *P. santacatalina*, *P. elvisi* sp. nov., and *P. sillardi* formed a grade with respect to a *P. goffrediae* sp. nov. and *P. orphanae* sp. nov. clade. The MP topology (Suppl. material 1: Fig. S2) differed in showing *P. elvisi* sp. nov. and *P. sillardi* as a clade (as opposed to a grade) that was sister group to the *P. goffrediae* sp. nov. and *P. orphanae* sp. nov. clade.

The uncorrected COI pairwise distances showed much higher interspecific distances than intraspecific distances for each proposed new species; the intraspecific COI distances ranged from 0–1.46%, while the interspecific COI distances ranged from 12.65–19.64% (Table 4). *Peinaleopolynoe orphanae* sp. nov. was least divergent (12.65%) to its sister taxon, *P. goffrediae* sp. nov. *Peinaleopolynoe goffrediae* sp. nov. (17.57%) and *P. orphanae* sp. nov. (18.63%) were the most divergent to *P. mineoi* sp. nov., which was their most distantly related taxon (Fig. 1).

**Table 4.** Minimum uncorrected interspecific pairwise distances for *Peinaleopolynoe* spp. COI data, generated with PAUP* v. 4.0. The maximum uncorrected intraspecific COI distances are shown as the bold diagonal. Distances marked with asterisk are discussed in the text.

		**1**	**2**	**3**	**4**	**5**	**6**
**1.**	***P. mineoi* sp. nov.**	**0.00440**	–	–	–	–	–
**2.**	***P. santacatalina***	0.16844	**0.00709**	–	–	–	–
**3.**	***P. elvisi* sp. nov.**	0.16520	0.17674	**0.01357**	–	–	–
**4.**	***P. sillardi***	0.18693	0.19636	0.16878	–	–	–
**5.**	***P. goffrediae* sp. nov.**	0.17570*	0.15659	0.15680	0.17423	**0.00000**	–
**6.**	***P. orphanae* sp. nov.**	0.18634*	0.15194	0.16170	0.18149	0.12645*	**0.01458**

### Haplotype networks

**Figure 2.**
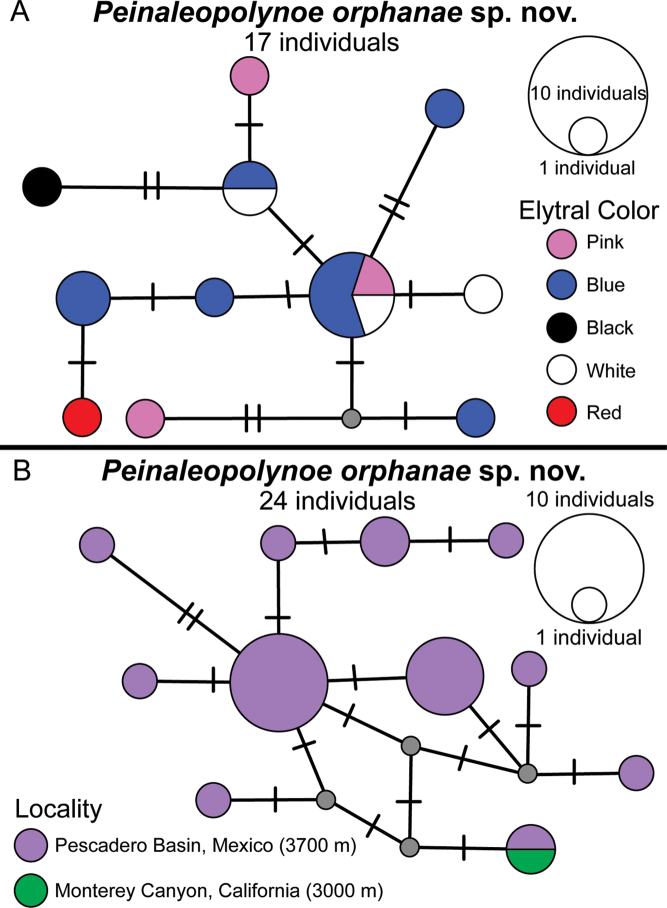
Haplotype networks from *P. orphanae* sp. nov. COI data, with small grey circles representing missing haplotypes **A** seventeen individuals with elytral color coding **B** twenty-four individuals with geographic locality coding, with twenty-three from the Pescadero Basin, Gulf of California, Mexico and one from Monterey Canyon, California.

The 17 specimens of *P. orphanae* sp. nov. that were collected with elytra remaining on the dorsum were coded for elytral color in the COI haplotype network (Fig. 2A), which displayed eleven distinct haplotypes. There was no correlation between elytral color and haplotype for the specimens with pink, blue, and white elytra; these specimens were spread amongst nine different haplotypes. Although the two specimens with red elytra and black elytra respectively had their own distinct haplotypes, there were only 17 total specimens analyzed, so it is unlikely this represents the full diversity of haplotypes. When the specimens of *P. orphanae* sp. nov. that had lost their elytra were included (*N* = 24 in total), there were eleven haplotypes (Fig. 2B) from the type locality of the Pescadero Basin. The single specimen from Monterey Canyon shared a haplotype with a Pescadero Basin specimen.

**Figure 3.**
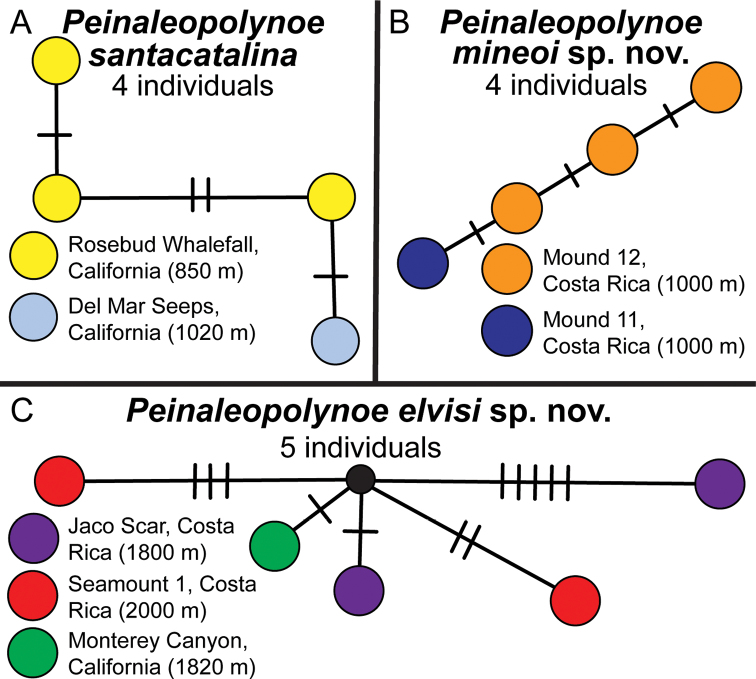
Haplotype networks from COI data with geographic locality coding; each colored circle represents a single individual **A***Peinaleopolynoe santacatalina* network includes four individuals, three from the Rosebud Whalefall off San Diego, California and one from the Del Mar Seeps, California **B***Peinaleopolynoe mineoi* sp. nov. network includes four individuals, three from Mound 12, Costa Rica and one from Mound 11, Costa Rica **C***Peinaleopolynoe elvisi* sp. nov. includes five individuals, two from Jaco Scar, Costa Rica, two from Seamount 1, Costa Rica, and one from the Patrick Whalefall in Monterey Canyon, California. The small black circle represents a missing haplotype.

For the four specimens of *P. santacatalina*, there were four haplotypes (Fig. 3A). For the four specimens of *P. mineoi* sp. nov., there were four haplotypes that differed from each other by only one base (Fig. 3B). Lastly, for the five specimens of *P. elvisi* sp. nov., there were five haplotypes across the three localities from Costa Rica to California (Fig. 3C).

### Character transformations

**Figure 4.**
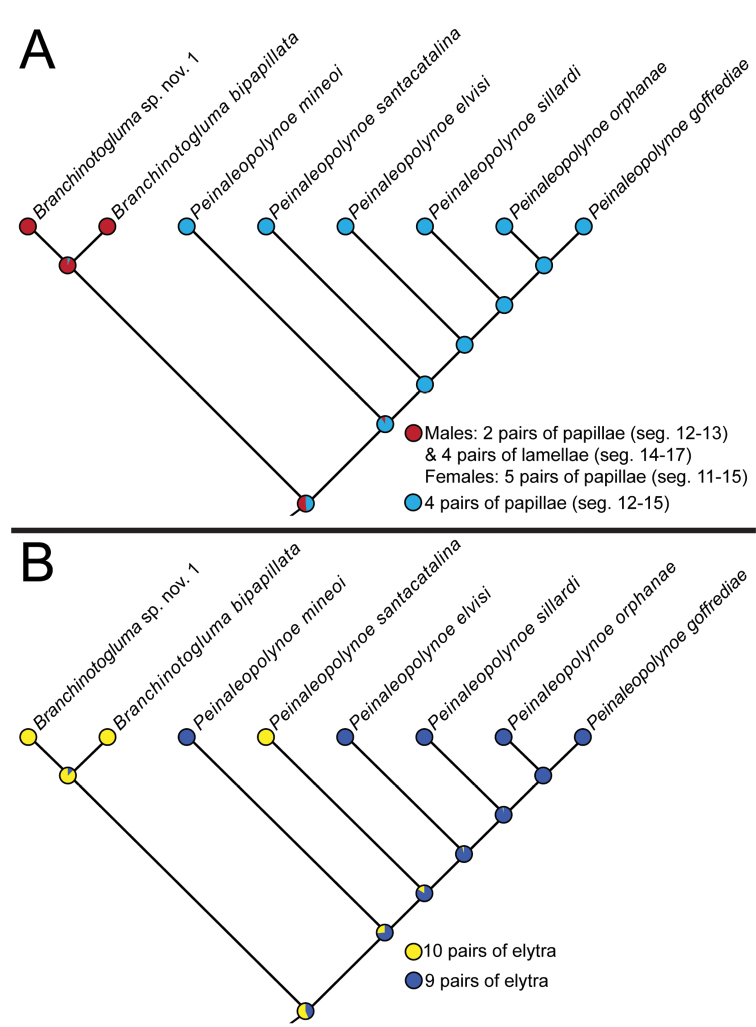
Maximum likelihood tree topology from concatenated data (COI, 16S, 18S, 28S, H3, CytB) for *Peinaleopolynoe* and its sister clade (*B. bipapillata* and *B.* sp. nov. 1), with the transformation for **A** ventral segmental papillae and/or lamellae and **B** elytral pairs. ‘Pie charts’ at the nodes represent probabilities for the relevant states.

The state for the *B.* sp. nov. 1 and *B. bipapillata* clade was males with two pairs of papillae on segments 12–13 and four pairs of lamellae on segments 14–17, and females with five pairs of papillae on segments 11–15. The ancestral state of ventral segmental papillae and/or lamellae was inferred to be four pairs of papillae present on segments 12–15 for *Peinaleopolynoe*, which is arguably also an apomorphy for the clade (Fig. 4A).

The ancestral state of elytra was unclear for *Peinaleopolynoe*, but there was a slightly greater likelihood of possessing nine pairs of elytra (Fig. 4B). A most parsimonious transformation under this scenario would imply a reversal to the outgroup state of ten pairs of elytra for *P. santacatalina* (Fig. 4B). The other equally parsimonious alternative would be for nine pairs of elytra to have evolved independently in *P. mineoi* sp. nov. and the clade comprised of *P. elvisi* sp. nov., *P. sillardi*, *P. goffrediae* sp. nov. and *P. orphanae* sp. nov.

## Taxonomy

### Polynoidae Kinberg, 1856

#### Lepidonotopodinae

Taxon classificationAnimaliaPhyllodocidaPolynoidae

Pettibone, 1983

7F87932E-B10A-5D34-AFFE-944367456F00

##### Diagnosis (emended).

Elytra and elytrophores range from seven to 12 pairs, on segments 2, 4, 5, 7, and the remaining odd segments. Prostomium with median antenna with ceratophore in anterior notch; eyes lacking; and a pair of tapering palps. Segment one with two pairs of tapering anterior cirri (= tentacular cirri). Parapodia biramous or sub-biramous. Notopodia with or without well-developed bracts; with or without branchiae, either plicate or arborescent if present. Dorsal cirri with cylindrical cirrophores present on non-elytrigerous segments. Ventral cirri with short tapering styles; segment 2 modified, with longer styles, directed anteriorly. Presence and placement of ventral segmental papillae variable.

##### Remarks.

Miura’s (1994) emended diagnosis of the subfamily Lepidonotopodinae is further emended to allow inclusion of several genera from an assemblage of original subfamilies: Macellicephalinae Hartmann-Schröder, 1971 (*Bathykurila* and *Levensteiniella*), Branchipolynoinae Pettibone, 1984 (*Branchipolynoe*), Branchiplicatinae Pettibone, 1985 (*Branchiplicatus*), and Branchinotogluminae Pettibone, 1985 (*Branchinotogluma* and *Peinaleopolynoe*), in addition to the previously included *Lepidonotopodium* and *Thermopolynoe*. It should be noted that Bonifácio and Menot (2018) emended Macellicephalinae to include Lepidonotopodinae, Branchipolynoinae, Branchiplicatinae, and Branchinotogluminae. The presence of notopodial bracts is no longer required for membership in this group. Genera may lack branchiae (*Bathykurila*, *Levensteiniella*, and *Lepidonotopodium*), as well as possess either parapodial plicate (*Branchiplicatus*) or arborescent branchiae (*Branchinotogluma*, *Peinaleopolynoe*, *Branchipolynoe*, and *Thermopolynoe*). Furthermore, we use the term anterior cirri as opposed to tentacular cirri, to clarify the position of cirri lying on segment 1 rather than the head (see Rouse and Pleijel 2001; Lindgren et al. 2019).

#### Peinaleopolynoe

Taxon classificationAnimaliaPhyllodocidaPolynoidae

Desbruyères & Laubier, 1988, emended

E50D1DE9-537C-5001-9035-0622561C685F

##### Type species.

*Peinaleopolynoe sillardi* Desbruyères & Laubier, 1988

##### Diagnosis (emended).

Twenty-one segments. Elytra large, sub-reniform, overlapping, and covering dorsum. Elytra with or without papillae and/or posterior extensions. Chaetae extending beyond the edge of elytra. Nine or ten pairs elytra and elytrophores on segments 2, 4, 5, 7, 9, 11, 13, 15, 17, 19, or lacking on 19. Pharynx with either seven pairs of border papillae, six pairs of border papillae, or seven dorsal and six ventral border papillae. Bilobed prostomium with triangular anterior lobes bearing lateral antennae (= minute frontal filaments, sensu Pettibone 1993). Median antenna in anterior notch. Paired palps. Eyes lacking. Achaetous segment 1 not visible dorsally and contains dorsal and ventral pairs of smooth, tapering anterior cirri (= tentacular cirri, sensu Pettibone 1993). Parapodia biramous. Neuropodia ranging from ca. twice the length to almost as long as notopodia. Dorsal tubercles, in line with elytrophores, on non-elytrigerous segments possessing small groups of branchiae. Notochaetae bundles stout. Neurochaetae long, slender. Dorsal cirri present on non-elytrigerous segments. In specimens with nine pairs elytra, segment 19 modified, lacking dorsal cirri. Cylindrical cirrophores and long distal styles (extending far beyond length of chaetae) of dorsal cirri. Arborescent branchiae beginning on segment 2 or 3 and continuing to near end of body. Branchiae attached on bases of notopodia and on dorsal tubercles. Four pairs of ventral segmental papillae on segments 12–15. Pygidium with a pair of anal cirri.

##### Remarks.

*Peinaleopolynoe* was erected for *P. sillardi* by Desbruyères and Laubier (1988), and subsequently emended by Pettibone (1993) to include *P. santacatalina*. Both species were found associated with organic falls on the seafloor, a whalefall in the case of *P. santacatalina*. Pettibone (1993) then placed the genus in the subfamily Branchinotogluminae, but the validity of this subfamily has been questioned (Bonifácio and Menot 2018). Pettibone’s diagnosis has been emended here to accommodate the inclusion of *P. orphanae* sp. nov., *P. elvisi* sp. nov., *P. goffrediae* sp. nov., and *P. mineoi* sp. nov. We present a table to compare the six different *Peinaleopolynoe* spp. (Table 5). Ventral papillae on segments 12–15 remains an apomorphy for this clade. However, arborescent branchiae may now begin on segment 2 or 3. The pharynx may now also possess six pairs of border papillae or seven dorsal and six ventral border papillae, in addition to the seven pairs of border papillae originally diagnosed. Pettibone (1993) noted a pair of minute frontal filaments on the triangular anterior lobes of the prostomium, which we prefer to refer to as lateral antennae. As in the emended diagnosis of Lepidonotopodinae, we use the term anterior cirri as opposed to tentacular cirri, to clarify the position of cirri lying on segment 1 rather than the head (see Rouse and Pleijel 2001; Lindgren et al. 2019).

**Table 5.** Morphological diagnostic characters of the six *Peinaleopolynoe* spp.

Characters	*P. sillardi*	*P. santacatalina*	*P. orphanae* sp. nov.	*P. elvisi* sp. nov.	*P. goffrediae* sp. nov.	*P. mineoi* sp. nov.
**Segments**	21	21	21	21	21	21
**Pharynx**	7 pairs of border papillae	7 pairs of border papillae	7 dorsal & 6 ventral border papillae	6 pairs of border papillae	7 dorsal & 6 ventral border papillae	7 dorsal & 6 ventral border papillae
**Elytra**	9 pairs: seg. 2, 4, 5, 7, 9, 11, 13, 15, 17	10 pairs: seg. 2, 4, 5, 7, 9, 11, 13, 15, 17, 19	9 pairs: seg. 2, 4, 5, 7, 9, 11, 13, 15, 17	9 pairs: seg. 2, 4, 5, 7, 9, 11, 13, 15, 17	9 pairs: seg. 2, 4, 5, 7, 9, 11, 13, 15, 17	9 pairs: seg. 2, 4, 5, 7, 9, 11, 13, 15, 17
**Macrotubercles on Elytra**	?	Few pointed on posterior margin	Few broad rounded on posterior margin	Single broad on posterior margin	Few pointed on posterior margin	Few broad rounded on posterior margin
**Branchiae**	Seg. 2-near the end of body	Seg. 2–20	Seg. 3–18	Seg. 3–16	Seg. 2–17	Seg. 3–16
**Modified Segments**	Seg. 19: lacking dorsal cirri and elytrophores	N/A	Seg. 19: lacking dorsal cirri and elytrophores	Seg. 19: lacking dorsal cirri and elytrophores	Seg. 19: lacking dorsal cirri and elytrophores	Seg. 19: lacking dorsal cirri and elytrophores
**Ventral Segmental Papillae**	4 pairs: seg. 12–15; relatively long, laterally curved	4 pairs: seg. 12–15; relatively long, laterally curved	4 pairs: seg. 12–15; small, rounded, cylindrical	4 pairs: seg. 12–15; relatively long, laterally curved	4 pairs: seg. 12–15; relatively long, laterally curved	4 pairs: seg. 12–15; relatively long, laterally curved
**Dorsal Cirri**	Seg. 3, 6, 8, 10, 12, 14, 16, 18, 20, 21	Seg. 3, 6, 8, 10, 12, 14, 16, 18, 20, 21	Seg. 3, 6, 8, 10, 12, 14, 16, 18, 20, 21	Seg. 3, 6, 8, 10, 12, 14, 16, 18, 20, 21	Seg. 3, 6, 8, 10, 12, 14, 16, 18, 20, 21	Seg. 3, 6, 8, 10, 12, 14, 16, 18, 20, 21
**Jaws**	Hooked with lateral teeth	Hooked with small and larger teeth on inner borders	Hooked with small teeth on inner borders	Hooked with small teeth on inner borders	Hooked with small teeth on inner borders	Hooked with large, rounded, protruding teeth on inner borders
**Angle of Neuroacicular Lobe**	Nearly horizontal	Nearly horizontal	Nearly horizontal	Nearly horizontal	Diagonal	Nearly horizontal
**Known Habitat**	Organic remains	Whalefalls, seep	Seep, hydrothermal vent microbial mat	Whalefall, deployed bone & wood	Whalefall	Deployed bone & wood

#### Peinaleopolynoe santacatalina

Taxon classificationAnimaliaPhyllodocidaPolynoidae

Pettibone, 1993

F33B0694-BC3C-5539-8B5D-D14F95C9D83E

Figure 5

##### Material examined.

Seven specimens (SIO-BIC A8487, SIO-BIC A8565, SIO-BIC A8566, SIO-BIC A8567, SIO-BIC A10927) from the Rosebud Whalefall off the coast of San Diego, California (32°46.62'N, 117°29.25'W), ROV “Doc Ricketts” dive 471, 842 m depth, 18 May 2013. One specimen (SIO-BIC A8487) fixed in formalin and preserved in 50% ethanol, with elytra fixed and preserved in 95%. Six specimens (SIO-BIC A8565, SIO-BIC A8566, SIO-BIC A8567, A10927) fixed in formalin and preserved in 50% ethanol. Twenty-eight specimens (SIO-BIC A8489, SIO-BIC A10924) from the Rosebud Whalefall (32°46.30'N, 117°27.18'W), ROV “Doc Ricketts” dive 623, 850 m depth, 20 June 2014. Three of the SIO-BIC A8489 specimens fixed and preserved in 95% ethanol, with elytra fixed and preserved in 95% ethanol; the remaining specimen fixed in formalin. Twenty-four specimens (SIO-BIC A10924) fixed and preserved in 95% ethanol. One specimen (SIO-BIC A8490) from the Del Mar Seeps off the coast of San Diego, California (32°54.25'N, 117°46.94'W), ROV “Doc Ricketts” dive 472, 1020 m depth, 19 May 2013; fixed in formalin and preserved in 50% ethanol, with elytra fixed and preserved in 95% ethanol.

**Figure 5.**
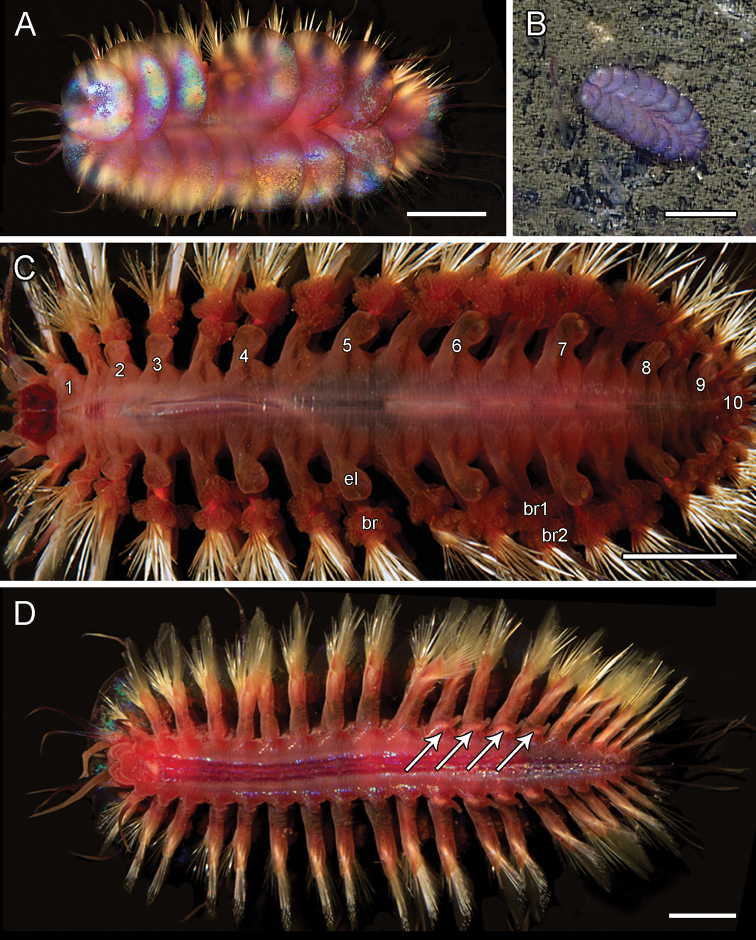
*Peinaleopolynoe santacatalina* specimens **A**SIO-BIC A8487, live dorsal view **B***Peinaleopolynoe santacatalina* observed on the Rosebud Whalefall off the coast of San Diego, CA **C**SIO-BIC A8489, live dorsal view without elytra. Numbers next to the elytrophores indicate the pairs of elytra (ten total) **D**SIO-BIC A10927, live ventral view. Arrows indicate the four pairs of papillae on segments 12–15. Abbreviations: el, elytrophore; br, single large group of branchiae on elytrigerous segment; br1, branchiae small group 1 attached to dorsal tubercle on cirrigerous segment; br2, branchiae large group 2 attached near base of notopodium on cirrigerous segment. Scale bars: 5 mm (**A, C, D**); 10 mm (**B**).

##### Supplementary description.

Elytra and elytrophores large, bulbous, ten pairs, on segments 2, 4, 5, 7, 9, 11, 13, 15, 17, 19. Elytra thin, smooth edges, oval-shaped with a very small sub-reniform notch at the anterior-facing edges. Elytra large, covering dorsum (Fig. 5A, B). Chaetae and dorsal cirri extending beyond the width of elytra (Fig. 5A). Elytra on segments 2, 17, 19 ca. half the size to three quarters of the size of mid-body elytra. Elytra on segments 17, 19 curving to a lateral point away from the midline (Fig. 5B). All specimens having lost some elytra in sampling process. Ten pairs of elytra confirmed by the presence of ten bulbous elytrophores, very small on segment 19 (Fig. 5C). Elytral color ranging from iridescent, light pink to deep red. Remaining morphological characters examined matching Pettibone’s (1993) original description, most importantly the presence of ventral papillae on segments 12–15 (Fig. 5D).

##### Remarks.

The specimens studied here were mostly collected from the San Diego whalefall (850 m depth), ca. 110 km southeast from the type locality whalefall (1240 m depth), and matched Pettibone’s (1993) description. One specimen, SIO-BIC A8490, was also collected at ~ 1000 m from a methane seep off Del Mar, southern California. *Peinaleopolynoe santacatalina* differs from the remaining *Peinaleopolynoe* taxa in that it has ten pairs of elytra as opposed to nine and no modified segment 19 (Table 5).

#### Peinaleopolynoe orphanae

Taxon classificationAnimaliaPhyllodocidaPolynoidae

Hatch & Rouse sp. nov.

0EAC0223-E07D-5D35-A084-7F283EFDDF9B

http://zoobank.org/759AD6A2-7695-42FC-9E33-0EF105A1D97A

**Figure 6.**
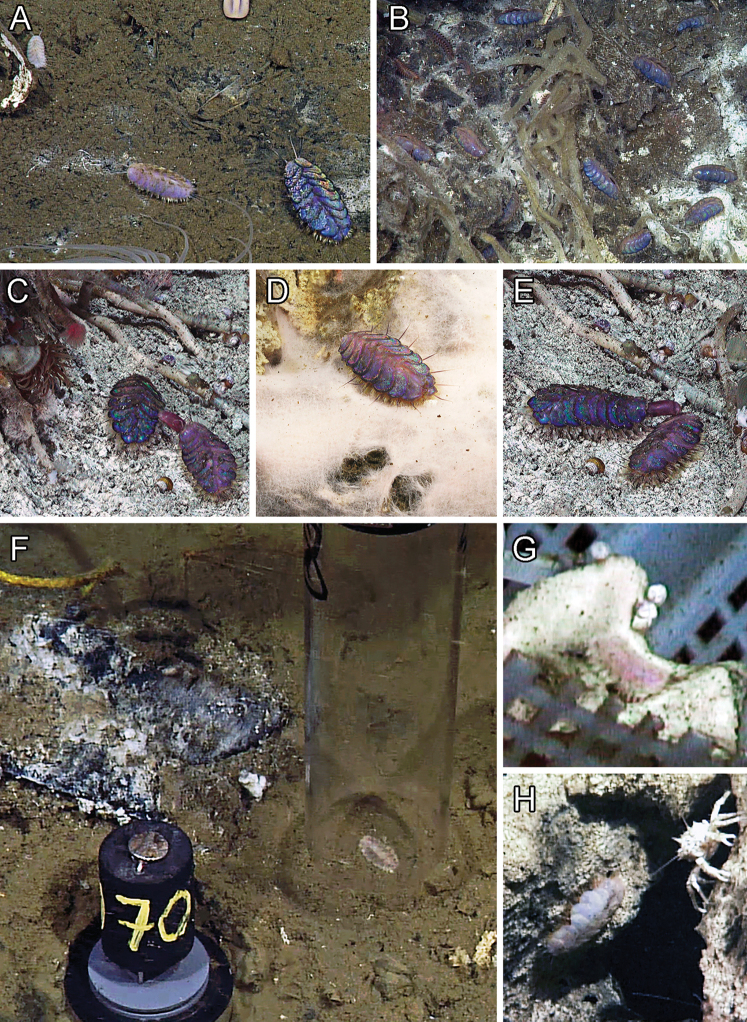
In situ photos of the new *Peinaleopolynoe* spp. **A–E***Peinaleopolynoe orphanae* sp. nov. observed in the Pescadero Basin, Gulf of California, Mexico: **C, E***Peinaleopolynoe orphanae* sp. nov. fighting behavior observed; the everted pharynx is used to bite off pieces of the opponent’s elytra **F***Peinaleopolynoe elvisi* sp. nov. holotype SIO-BIC A8488 observed and collected next to a whale bone in the Monterey Canyon, California **G***Peinaleopolynoe elvisi* sp. nov. paratype SIO-BIC A9699 observed and collected on a pig bone deployment from Jaco Scar, Costa Rica **H***Peinaleopolynoe goffrediae* sp. nov. holotype SIO-BIC A5485 observed and collected on a whalefall in the Monterey Canyon, California.

**Figure 7.**
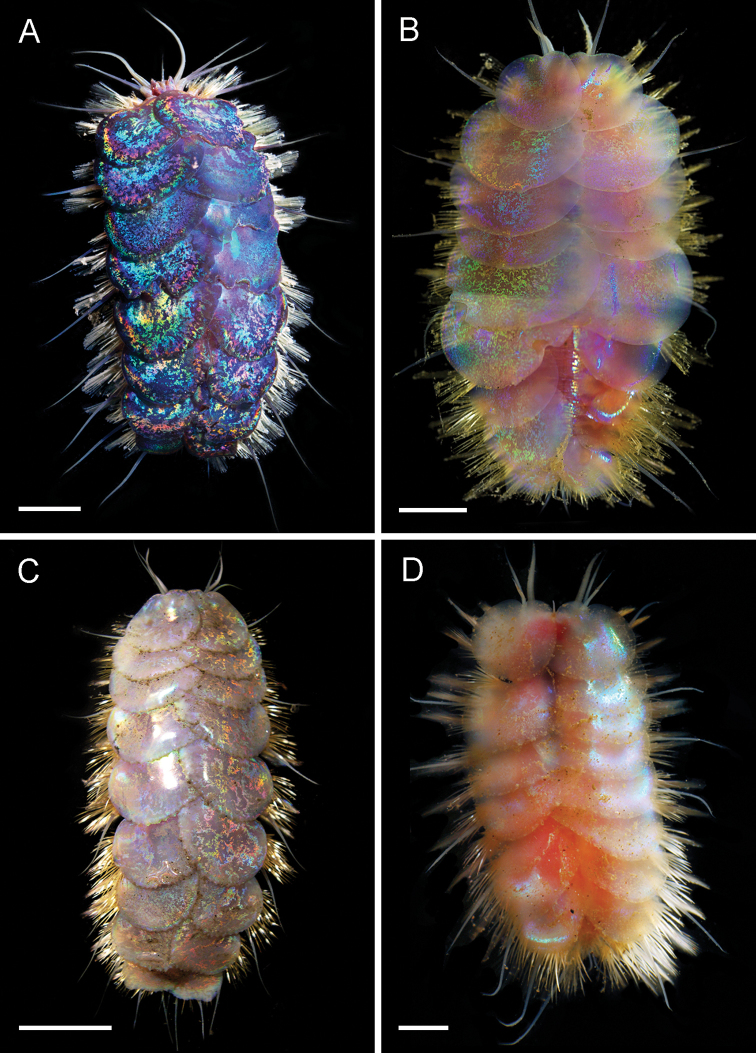
Live dorsal views of the new *Peinaleopolynoe* spp. **A***Peinaleopolynoe orphanae* sp. nov. holotype SIO-BIC A6151 **B***Peinaleopolynoe elvisi* sp. nov. holotype SIO-BIC A8488 **C***Peinaleopolynoe goffrediae* sp. nov. holotype SIO-BIC A5485 **D***Peinaleopolynoe mineoi* sp. nov. holotype SIO-BIC A10071. Scale bars: 6 mm (**A**); 8 mm (**B, C**); 1 mm (**D**).

**Figure 8.**
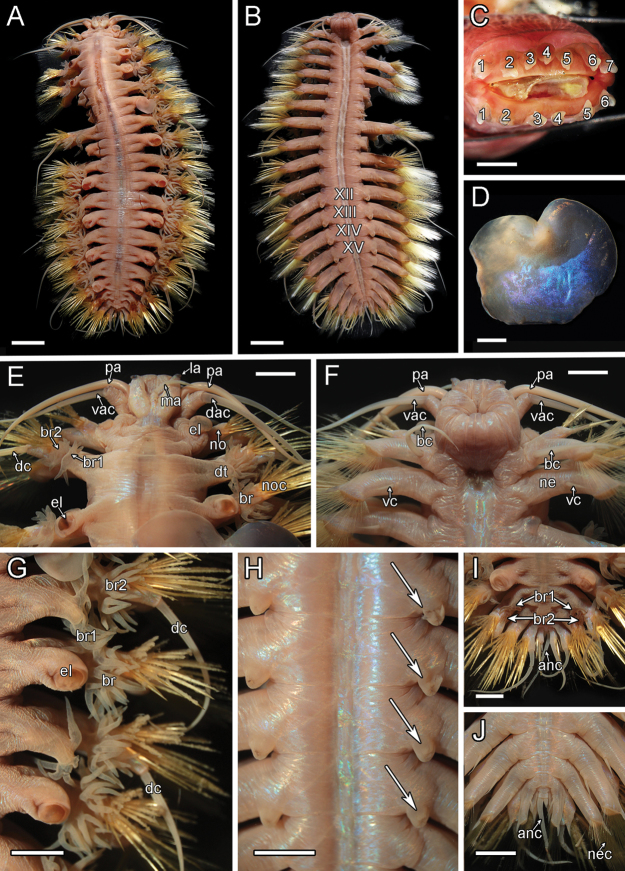
Macro photos and micrographs of *P. orphanae* sp. nov. holotype SIO-BIC A6151 and paratype SIO-BIC A9996 **A** dorsal view, holotype **B** ventral view, holotype. Segments 12–15 are marked to indicate the presence of four pairs of papillae **C** frontal view of proboscis showing papillae, paratype. Numbers mark the papillae on the dorsal (seven papillae) and ventral (six papillae) surfaces **D** right elytron from segment 5, holotype **E** dorsal view of anterior, holotype **F** ventral view of anterior, holotype **G** right side branchiae on segments 8–11, holotype **H** ventral papillae on segments 12–15 (four pairs) indicated by arrows, holotype **I** dorsal view of posterior, holotype **J** ventral view of posterior, holotype. Abbreviations: XII, segment 12; XIII, segment 13; XIV, segment 14; XV, segment 15; ma, median antenna; la, lateral antenna; pa, palp; dac, dorsal anterior cirrus; vac, ventral anterior cirrus; el, elytrophore; br, single large group of branchiae on elytrigerous segment; noc, notochaetae; dc, dorsal cirrus; vc, ventral cirrus; bc, buccal cirrus; br1, branchiae small group 1 attached to dorsal tubercle on cirrigerous segment; br2, branchiae large group 2 attached near base of notopodium on cirrigerous segment; no, notopodium; ne, neuropodium; nec, neurochaetae; dt, dorsal tubercle; anc, anal cirrus. Scale bars: 4 mm (**A, B**); 2 mm (**C–J**).

**Figure 9.**
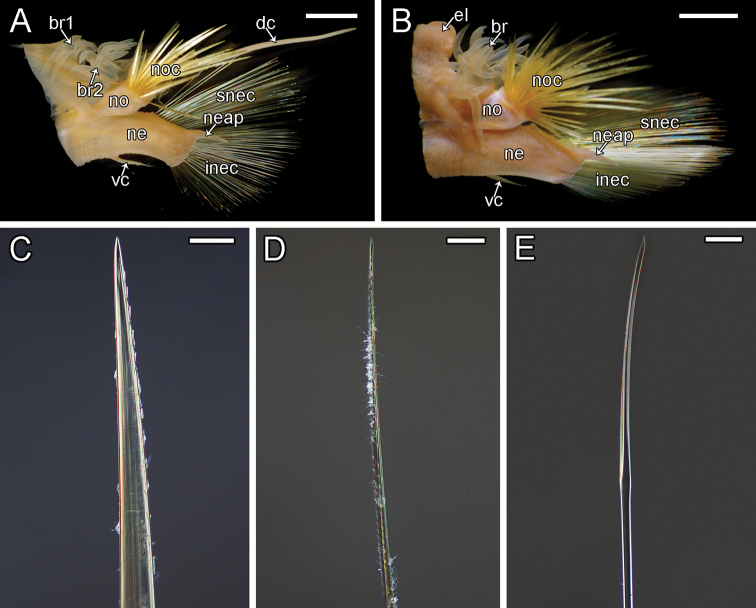
Micrographs of *P. orphanae* sp. nov. holotype SIO-BIC A6151 **A** left parapodium from segment 6 **B** left parapodium from segment 9 **C** notochaeta **D** superior neurochaeta (supra-acicular) **E** inferior neurochaeta (subacicular). Abbreviations: br1, branchiae small group 1 attached to dorsal tubercle; br2, branchiae large group 2 attached near base of notopodium; noc, notochaetae; dc, dorsal cirrus; no, notopodium; snec, superior neurochaetae; ne, neuropodium; neap, neuroacicular process; vc, ventral cirrus; inec, inferior neurochaetae; el, elytrophore; br, single large group of branchiae on elytrigerous segment. Scale bars: 2 mm (**A, B**); 15 μm (**C**); 10 μm (**D, E**).

**Figure 10.**
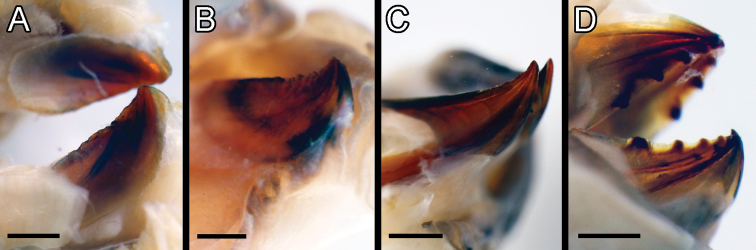
Micrographs of *Peinaleopolynoe* spp. jaws **A***Peinaleopolynoe orphanae* sp. nov. paratype SIO-BIC A9996 **B***Peinaleopolynoe elvisi* sp. nov. holotype SIO-BIC A8488 **C***Peinaleopolynoe goffrediae* sp. nov. holotype SIO-BIC A5485 **D***Peinaleopolynoe mineoi* sp. nov. paratype SIO-BIC A9709. Scale bars: 3 mm (**A, C**); 2 mm (**B, D**).

**Figure 11.**
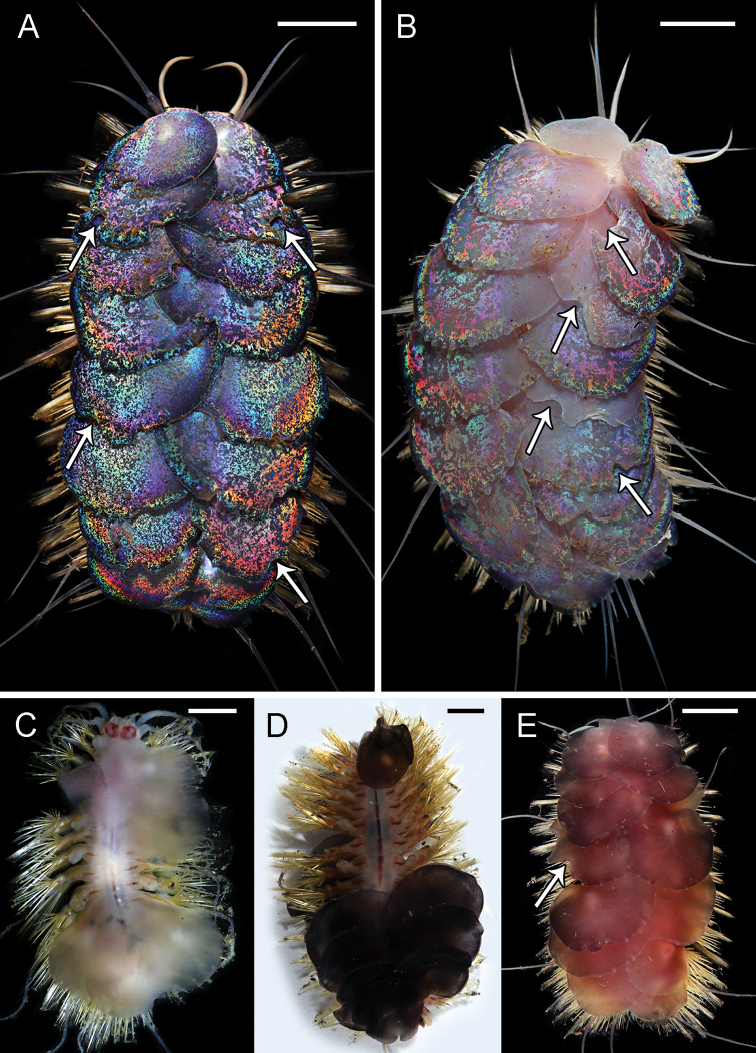
Live dorsal views of *P. orphanae* sp. nov. Arrows indicate elytral bite marks from the fighting behavior **A** paratype SIO-BIC A10020 with blue elytra **B** paratype SIO-BIC A10024 with pink elytra **C** paratype SIO-BIC A6312 with white elytra **D** paratype SIO-BIC A6166 with black elytra **E** paratype SIO-BIC A10023 with red elytra. Scale bars: 5 mm (**A, B, D, E**); 3 mm (**C**).

Figures 6A–E , 7A , 8 , 9 , 10A , 11

Peinaleopolynoe sp. nov. 1 Goffredi et al., 2017.

##### Type locality.

Hydrothermal vents of the Pescadero Basin in the Gulf of California, Mexico (23°57.23'N, 108°51.73'W), ROV “Doc Ricketts” Dive 757, 3700 m depth, 24 April 2015.

##### Material examined.

**Type specimen: *Holotype*** (SIO-BIC A6151) from the Pescadero Basin in the Gulf of California, Mexico (23°57.23'N, 108°51.73'W), ROV “Doc Ricketts” Dive 757, 3700 m depth, 24 April 2015; fixed in formalin and preserved in 50% ethanol, with elytra fixed and preserved in 95% ethanol. ***Paratypes***: Two specimens (SIO-BIC A8597 and SIO-BIC A6166) from the same location as holotype; both fixed in formalin and preserved in 50% ethanol, with elytra fixed and preserved in 95% ethanol. Five specimens (SIO-BIC A6150, SIO-BIC A6155, SIO-BIC A6163, UNAM-ICML-EMU-12666) from the Pescadero Basin in the Gulf of California, Mexico (24°0'N, 108°49.98'W), ROV “Doc Ricketts” Dive 750, 3676 m depth, 18 April 2015; all five specimens fixed in formalin and preserved in 50% ethanol, with elytra fixed and preserved in 95% ethanol. One specimen (SIO-BIC A6312) from the Pescadero Basin in the Gulf of California, Mexico (24°0.00'N, 108°49.98'W), ROV “Doc Ricketts” Dive 751, 3676 m depth, 19 April 2015; fixed in formalin and preserved in 50% ethanol, with elytra fixed and preserved in 95% ethanol. One specimen (SIO-BIC A9989) from the Pescadero Basin in the Gulf of California, Mexico (23°57.37'N, 108°51.71'W), ROV “SuBastian” Dive S0196, 3688 m depth, 17 November 2018; fixed and preserved in 95% ethanol. One specimen (SIO-BIC A9988) from the Pescadero Basin in the Gulf of California, Mexico (23°57.41'N, 108°51.82'W), ROV “SuBastian” Dive S0196, 3670 m depth, 17 November 2018; fixed in formalin and preserved in 50% ethanol, with elytra fixed and preserved in 95% ethanol. Four specimens (SIO-BIC A10922, SIO-BIC A10921, SIO-BIC A10923, SIO-BIC A10003) from the Pescadero Basin in the Gulf of California, Mexico (23°56.51'N, 108°51.34'W), ROV “SuBastian” Dive S0197, 3692 m depth, 18 November 2018; all four specimens fixed in 95% ethanol and preserved in 50% ethanol. Two specimens (SIO-BIC A10001, SIO-BIC A9996) from the Pescadero Basin in the Gulf of California, Mexico (23°56.49'N, 108°51.35'W), ROV “SuBastian” Dive S0197, 3666–3667 m depth, 18 November 2018; one fixed in formalin and preserved in 50% ethanol, with elytra fixed and preserved in 95% ethanol, and one fixed in 95% ethanol and preserved in 50% ethanol. Eight specimens (SIO-BIC A10021, SIO-BIC A10025, SIO-BIC A10037, SIO-BIC A10020, SIO-BIC A10026, SIO-BIC A10022, SIO-BIC A10023, SIO-BIC A10024, SIO-BIC A10036) from the Pescadero Basin in the Gulf of California, Mexico (23°57.37'N, 108°51.71'W), ROV “SuBastian” Dive S0200, 3687–3688 m depth, 21 November 2018; three fixed in formalin and preserved in 50% ethanol, with elytra fixed and preserved in 95% ethanol, one fixed in 95% ethanol and preserved in 50% ethanol, and four fixed and preserved in 95% ethanol. One specimen (SIO-BIC A10926) from a vesicomyid clam bed near a whalefall in the Monterey Canyon, California (36°46.33'N, 122°4.99'W), ROV “Doc Ricketts” Dive 208, ~2900 m depth, 28 October 2010; fixed and preserved in 95% ethanol.

##### Description.

In life, large, overlapping, iridescent blue elytra covering the dorsum. Dorsum with ciliated transverse bands extending onto bases of elytrophores and dorsal tubercles. Chaetae extending beyond the width of elytra (Figs 6A–E, 7A, 8A). Twenty-one segments total (Fig. 8A, B). Elytra and elytrophores large, bulbous, nine pairs, on segments 2, 4, 5, 7, 9, 11, 13, 15, 17 (Fig. 8A). Elytra sub-reniform, thick, and greatly textured with large, bulbous macrotubercles along posterior margin (Figs 7A, 8D, 10B, E). Elytra on segment 17 curve to a lateral point in live specimen (Fig. 7A). Pharynx with seven dorsal border papillae and six ventral border papillae (Fig. 8C). Bilobed prostomium with triangular anterior lobes bearing short, thin, very delicate lateral antennae (= minute frontal filaments, sensu Pettibone 1993). Smooth median antenna with bulbous ceratophore in anterior notch. Eyes lacking. Pair of thick, smooth, tapering palps, ca. three times the length of prostomium (Fig. 8E). Segment 1 with dorsal and ventral pairs of smooth, tapering anterior cirri (= tentacular cirri, sensu Pettibone 1993), ca. the same length as palps. Ventral anterior cirri slightly shorter than dorsal anterior cirri. Cirrophores of anterior cirri long and cylindrical, each with small acicular lobe on inner side (Fig. 8E, F). Smooth ventral cirri on segments 2–21 (Figs 8B, 9A, B). Buccal cirri of segment 2 modified, with bulbous ceratophores and longer styles, ca. four times the length of remaining ventral cirri (Fig. 8F). Buccal cirri attached to base of neuropodia. Ventral cirri on segments 3–21 attached to middle of neuropodia, with bulbous ceratophores and short, tapering styles. Dorsal cirri present on non-elytrigerous segments 3, 6, 8, 10, 12, 14, 16, 18, 20, 21 (Fig. 8A). Cirrophores of dorsal cirri cylindrical, rather long, fused to posterior sides of notopodia. Styles of dorsal cirri long, extending far beyond length of chaetae, filiform, tapering to fine tips. Segment 19 modified, lacking dorsal cirri and elytrophores (Fig. 8I). Arborescent branchiae compact, with numerous long terminal filaments, beginning on segment 3 (Fig. 8E) and continuing to segment 18 (Fig. 8I). Branchiae forming single large groups on elytrigerous segments, attached on bases of notopodia. Branchiae forming two groups on cirrigerous segments; small groups attached to dorsal tubercles and large groups attached near bases of notopodia (Fig. 8G). Nine pairs of thin rounded folds of unknown function attached to anterior sides of neuropodia on segments 4–12. On the right side, three additional thin rounded folds are visible on segments 13–15. Four pairs of ventral segmental papillae on segments 12–15 (Fig. 8B); small, rounded, and slightly cylindrical (Fig. 8H). Pygidium with a pair of anal cirri, not extending beyond the outline of the body (Fig. 8J). Parapodia biramous. Neuropodia ca. twice the length of notopodia, with an acicular process. On cirrigerous segments, notopodia with dorsal tubercles possessing small bundles of branchiae (Fig. 9A, B). Notopodia extending distally into acicular processes. Notochaetae in radiating bundles, stout, with double rows of spines (Fig. 9C); almost as long as neurochaetae. Neurochaetae slender, forming fan-shaped bundles (Fig. 9A, B). Superior neurochaetae (supra-acicular) with double rows of spines, and slightly curved tips (Fig. 9D). Inferior neurochaetae (sub-acicular) with double rows of teeth from the mid swelling to the hooked tips; smooth beneath the mid swelling (Fig. 9E). Inferior neurochaetae teeth are less prominent than the superior neurochaetae spines. Hooked jaws with small teeth on inner borders (Fig. 10A).

##### Morphological variation.

The holotype is 48 mm long, 27 mm wide, including chaetae. Smallest paratype (SIO-BIC A6312) is 21 mm long, 9 mm wide, including chaetae. Remaining paratypes range from 31–45 mm long, 18–26 mm wide, including chaetae. Paratypes vary in elytral color. Of the specimens examined, 19 individuals were collected with elytra remaining on the dorsum; the remaining specimens lost their elytra during the sampling process. SIO-BIC A8597, SIO-BIC A6155, SIO-BIC A10921, SIO-BIC A9996, SIO-BIC A10003, SIO-BIC A10923, SIO-BIC A10922, SIO-BIC A10021, and SIO-BIC A10020 (Fig. 11A) had the same iridescent blue elytra as the holotype. UNAM-ICML-EMU-12666, SIO-BIC A6150, SIO-BIC A10022, and SIO-BIC A10024 (Fig. 11B) had iridescent pink elytra; SIO-BIC A6312 (Fig. 11C), SIO-BIC A10025, and SIO-BIC A10001 had iridescent white elytra; SIO-BIC A6166 had iridescent black elytra (Fig. 11D); and SIO-BIC A10023 had red elytra (Fig. 11E). Some paratypes possess two pairs of ventral lamellae on segments 16–17 following the four pairs of ventral papillae on segments 12–15. Rounded ventral lamellae have similar orientation as papillae but flattened and not protruding as much toward posterior end. This dimorphism may be sexual, but it is presently unclear which sex is which.

##### Remarks.

*Peinaleopolynoe orphanae* sp. nov. is unique from the remaining *Peinaleopolynoe* taxa in that branchiae end on segment 18 (Table 5). Additionally, *P. orphanae* sp. nov. differs from its closest relative *P. goffrediae* sp. nov. in having small, rounded ventral papillae, as opposed to relatively long, laterally curved ventral papillae. The branchiae distributions range from segments 3–18 in *P. orphanae* sp. nov. but are present on segments 2–17 in *P. goffrediae* sp. nov.

##### Etymology.

*Peinaleopolynoe orphanae* sp. nov. is named after Dr. Victoria J. Orphan, not only for her invaluable research on deep-sea microorganisms, but also for her exploration of deep-sea chemosynthetic ecosystems and her love of the animals that thrive there.

##### Ecology.

*Peinaleopolynoe orphanae* sp. nov. is unusual among *Peinaleopolynoe* in that most specimens were associated with bacterial mats adjacent to hydrothermal vents in the Pescadero Basin at ~3700 m depth. One specimen (SIO-BIC A10926) was found at a cold seep with abundant vesicomyid clams suggesting that *P. orphanae* sp. nov. may be more of a habitat generalist than its close relatives.

*Peinaleopolynoe orphanae* sp. nov. displayed an interesting fighting behavior *in situ* (Fig. 6C, E), in which an individual used its everted pharynx to attack an opponent’s elytra; the two individuals attacked one another back and forth for several minutes (Suppl. material 2: movie). This may explain the damaged elytra with apparent bite marks on the posterior edges in the holotype (Fig. 7A) and in several other paratypes collected (Fig. 11A, B, E).

#### Peinaleopolynoe elvisi

Taxon classificationAnimaliaPhyllodocidaPolynoidae

Hatch & Rouse sp. nov.

EBC25AA1-E927-5ECB-AAE9-DC22C6326B56

http://zoobank.org/8F4C61A7-630F-4512-8DE8-AE62A2D374A5

**Figure 12.**
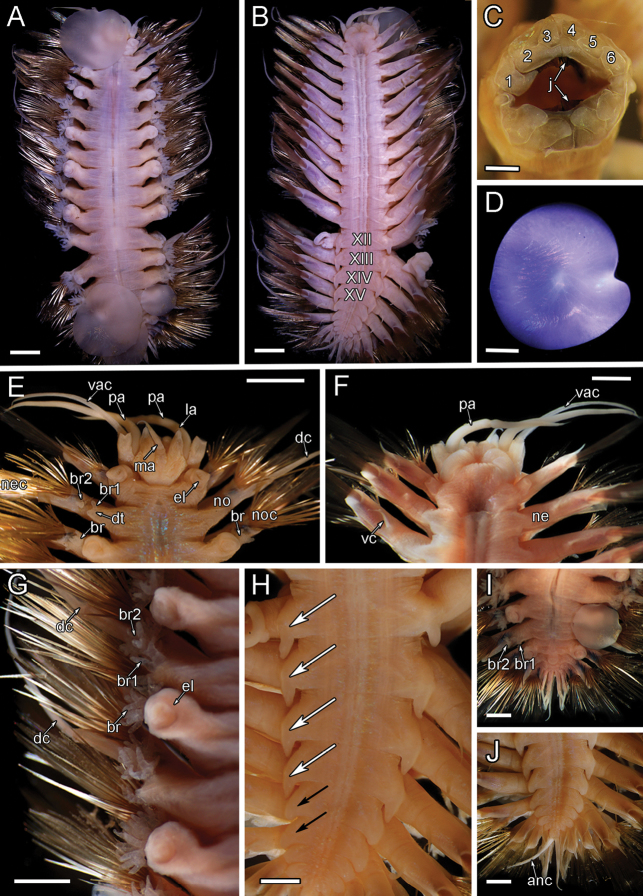
Macro photos and micrographs of *P. elvisi* sp. nov. holotype SIO-BIC A8488 **A** dorsal view **B** ventral view. Segments 12–15 are marked to indicate the presence of four pairs of papillae **C** frontal view of proboscis showing papillae and jaws. Numbers mark the papillae on the dorsal (six papillae) surface **D** left elytron from segment 2 **E** dorsal view of anterior **F** ventral view of anterior **G** left side branchiae on segments 7–11 **H** ventral papillae on segments 12–15 (four pairs) indicated by white arrows. Ventral lamellae on segments 16–17 (two pairs) indicated by black arrows **I** dorsal view of posterior **J** ventral view of posterior. Abbreviations: XII, segment 12; XIII, segment 13; XIV, segment 14; XV, segment 15; j, jaws; ma, median antenna; pa, palp; la, lateral antenna; vac, ventral anterior cirrus; el, elytrophore; br, single large group of branchiae on elytrigerous segment; noc, notochaetae; dc, dorsal cirrus; br1, branchiae small group 1 attached to dorsal tubercle on cirrigerous segment; br2, branchiae large group 2 attached near base of notopodium on cirrigerous segment; no, notopodium; nec, neurochaetae; vc, ventral cirrus; ne, neuropodium; dt, dorsal tubercle; anc, anal cirrus. Scale bars: 4 mm (**A, B**); 0.5 mm (**C**); 1 mm (**D, H–J**); 2 mm (**E–G**).

**Figure 13.**
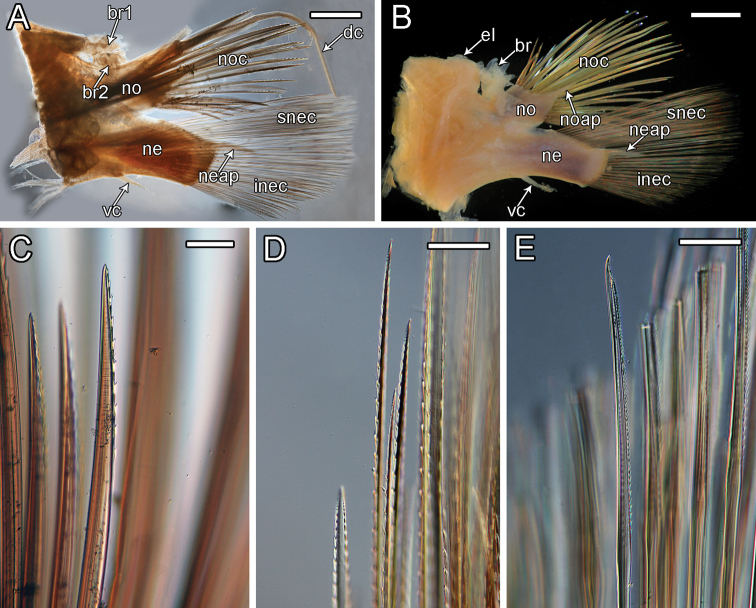
Micrographs of *P. elvisi* sp. nov. holotype SIO-BIC A8488 **A** right parapodium from segment 10 **B** right parapodium from segment 7 **C** notochaetae **D** superior neurochaetae (supra-acicular) **E** inferior neurochaetae (subacicular). Abbreviations: br1, branchiae small group 1 attached to dorsal tubercle; br2, branchiae large group 2 attached near base of notopodium; noc, notochaetae; dc, dorsal cirrus; no, notopodium; snec, superior neurochaetae; ne, neuropodium; neap, neuroacicular process; noap, notoacicular process; vc, ventral cirrus; inec, inferior neurochaetae; el, elytrophore; br, single large group of branchiae on elytrigerous segment. Scale bars: 1 mm (**A, B**); 15 μm (**C**); 10 μm (**D, E**).

Figures 6F–G , 7B , 10B , 12 , 13

##### Type locality.

Whalefall in Monterey Canyon, California (36°46.33'N, 122°4.99'W), ROV “Doc Ricketts” Dive 99, 1820 m depth, 20 November 2009.

##### Material examined.

**Type specimen: *Holotype*** (SIO-BIC A8488) from a whalefall in Monterey Canyon, California (36°46.33'N, 122°4.99'W), ROV “Doc Ricketts” Dive 99, 1820 m depth, 20 November 2009; fixed in formalin and preserved in 50% ethanol, with elytra fixed and preserved in 95% ethanol. ***Paratypes***: One specimen (SIO-BIC A9699) from bones deployed at Jaco Scar, Costa Rica (9°6.88'N, 84°50.14'W), HOV “Alvin” Dive AD4972, 1845 m depth, 18 October 2018; fixed in formalin and preserved in 50% ethanol, with elytra fixed and preserved in 95% ethanol. One specimen (MZUCR 1000-01) from bones deployed at Jaco Scar (9°6.91'N, 84°50.39'W), HOV “Alvin” Dive AD4976, 1887 m depth, 22 October 2018; fixed in formalin and preserved in 50% ethanol, with elytra fixed and preserved in 95% ethanol. Two specimens (SIO-BIC A9871, SIO-BIC A9870) from bones deployed at Seamount 1, Costa Rica (8°52.60'N, 85°7.34'W), HOV “Alvin” Dive AD4983, 2091 m depth, 29 October 2018; one fixed in formalin and preserved in 50% ethanol, with elytra fixed and preserved in 95% ethanol, and one fixed and preserved in 95% ethanol.

##### Description.

In life, large, overlapping, semi-transparent, iridescent pink elytra covering the dorsum. Dorsum with ciliated transverse bands extending onto bases of elytrophores and dorsal tubercles. Chaetae extending beyond the width of elytra (Fig. 7B). Twenty-one segments total (Fig. 12A, B). Elytra and elytrophores large, bulbous, nine pairs, on segments 2, 4, 5, 7, 9, 11, 13, 15, 17 (Fig. 12A). Elytra rounded to oval-shaped and slightly sub-reniform, very thin. Smooth edges along the circumference of elytra, except for a single rounded broad macrotubercle on posterior margin of elytra on segments 2, 4, 5, 7, 9, 11, 13, 15. Elytra on segments 2, 15, 17 ca. half the size of mid-body elytra. Elytra on segment 17 curving to a lateral point in live specimen (Figs 7B, 12D). Pharynx with a total of six dorsal and six ventral border papillae (Fig. 12C). Bilobed prostomium with triangular anterior lobes bearing short, thin, very delicate lateral antennae (= minute frontal filaments, sensu Pettibone 1993). Smooth median antenna with bulbous ceratophore in anterior notch. Eyes lacking. Pair of thick, smooth, tapering palps, ca. three times the length of prostomium (Fig. 12E). Segment 1 with dorsal and ventral pairs of smooth, tapering anterior cirri (= tentacular cirri, sensu Pettibone 1993), ca. the same length as palps. Ventral anterior cirri slightly shorter than dorsal anterior cirri. Cirrophores of anterior cirri long and cylindrical, each with small acicular lobe on inner side (Fig. 12E, F). Smooth ventral cirri on segments 2–21. Buccal cirri of segment 2 modified, with bulbous ceratophores (Fig. 12F) and longer styles. Buccal cirri attached to base of neuropodia. Ventral cirri on segments 3–21 attached to middle of neuropodia, with bulbous ceratophores and short, tapering styles (Fig. 12B). Dorsal cirri present on non-elytrigerous segments 3, 6, 8, 10, 12, 14, 16, 18, 20, 21 (Fig. 12A). Cirrophores of dorsal cirri cylindrical, rather long, fused to posterior sides of notopodia. Styles of dorsal cirri filiform, long, extending beyond length of chaetae. Segment 19 modified, lacking dorsal cirri and elytrophores (Fig. 12I). Arborescent branchiae compact, with numerous short, bulbous terminal filaments, beginning on segment 3 (Fig. 12E) and continuing to segment 16 (Fig. 12I). Branchiae forming single large groups on elytrigerous segments, attached to bases of notopodia. Branchiae forming two groups on cirrigerous segments; small groups attached to dorsal tubercles and large groups attached near bases of notopodia (Fig. 12G). Branchiae on segment 3 not fully developed, but formation of two distinct bundles of branchiae still apparent on the left side (Fig. 12E). Four pairs of ventral segmental papillae on segments 12–15 (Fig. 12B). Ventral papillae rather long, slender, curved laterally and followed by two pairs of ventral lamellae (Fig. 12H). Rounded ventral lamellae have similar orientation as papillae but flattened and not protruding as much toward posterior end. Pygidium with a pair of anal cirri extending to approximately the outline of the body (Fig. 12J). Parapodia biramous. Neuropodia ca. twice the length of notopodia, with an acicular process. On cirrigerous segments, notopodia with dorsal tubercles possessing small bundles of branchiae (Fig. 13A, B). Notopodia extending distally into acicular processes. Notochaetae forming radiating bundles, stout, with double rows of spines (Fig. 13C). Notochaetae almost as long as neurochaetae (Fig. 13A, B). Neurochaetae slender, forming fan-shaped bundles (Fig. 13A, B). Superior neurochaetae (supra-acicular) with double rows of spines (Fig. 13D). Inferior neurochaetae (sub-acicular) with double rows of teeth from the mid swelling to the hooked tips; smooth beneath the mid swelling (Fig. 13E). Inferior neurochaetae teeth are less prominent than the superior neurochaetae spines. Hooked jaws with small teeth on inner borders (Fig. 10B).

##### Morphological variation.

The holotype is 26 mm long, 15 mm wide, including chaetae. Paratypes range from 10–17 mm long, 7–9 mm wide, including chaetae.

##### Remarks.

*Peinaleopolynoe elvisi* sp. nov. is unique from the remaining *Peinaleopolynoe* taxa in having six pairs of border papillae on the pharynx (Table 5). Additionally, *P. elvisi* sp. nov. differs from its closest relatives *P. santacatalina* and *P. sillardi* in having branchiae start on segment 3, as opposed to on segment 2. Finally, the posterior margin of the elytra displays a single macrotubercle compared to the few found in the other species.

##### Etymology.

*Peinaleopolynoe elvisi* sp. nov. is named after the legendary King of Rock and Roll, Elvis Presley; the iridescent golden/pink elytra are reminiscent of the sparkly, sequined costumes he favored in his late career.

##### Ecology.

All specimens of *P. elvisi* sp. nov. were found associated with vertebrate bones or wood (Table 5). Fig. 6F shows the holotype observed *in situ* on sediment next to a whalefall just before collection. Fig. 6G shows paratype SIO-BIC A9699 observed *in situ* on a deployed pig bone before collection.

#### Peinaleopolynoe goffrediae

Taxon classificationAnimaliaPhyllodocidaPolynoidae

Hatch & Rouse sp. nov.

5DE2D1D0-2FDD-50AE-BB5D-4E3E9AC32F08

http://zoobank.org/19C2BB3E-1CE6-4341-8071-9CF82E9AC703

**Figure 14.**
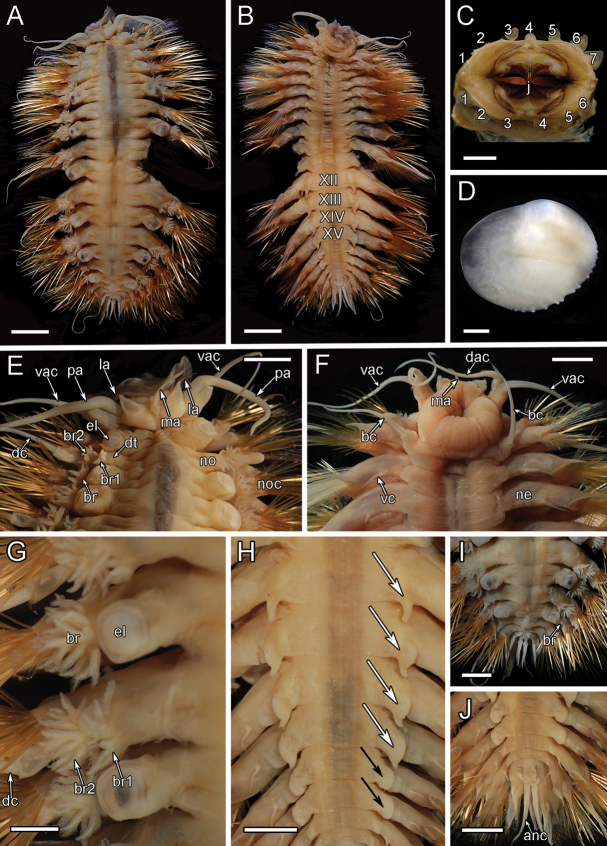
Macro photos and micrographs of *P. goffrediae* sp. nov. holotype SIO-BIC A5485 and paratype SIO-BIC A5464 **A** dorsal view, holotype **B** ventral view, holotype. Segments 12–15 are marked to indicate the presence of four pairs of papillae **C** frontal view of proboscis showing papillae and jaws, holotype. Numbers mark the papillae on the dorsal (seven papillae) and ventral (six papillae) surfaces **D** loose elytron, holotype **E** dorsal view of anterior, holotype **F** ventral view of anterior, paratype **G** left side branchiae on segments 12–15, holotype **H** ventral papillae on segments 12–15 (four pairs) indicated by white arrows, holotype. Ventral lamellae on segments 16–17 (two pairs) indicated by black arrows, holotype **I** dorsal view of posterior, holotype **J** ventral view of posterior, holotype. Abbreviations: XII, segment 12; XIII, segment 13; XIV, segment 14; XV, segment 15; j, jaws; ma, median antenna; la, lateral antenna; pa, palp; dac, dorsal anterior cirrus; vac, ventral anterior cirrus; el, elytrophore; br, single large group of branchiae on elytrigerous segment; noc, notochaetae; dc, dorsal cirrus; br1, branchiae small group 1 attached to dorsal tubercle on cirrigerous segment; br2, branchiae large group 2 attached near base of notopodium on cirrigerous segment; no, notopodium; vc, ventral cirrus; bc, buccal cirrus; ne, neuropodium; dt, dorsal tubercle; anc, anal cirrus. Scale bars: 4 mm (**A, B**); 2 mm (**C–F, H–J**); 1 mm (**G**).

**Figure 15.**
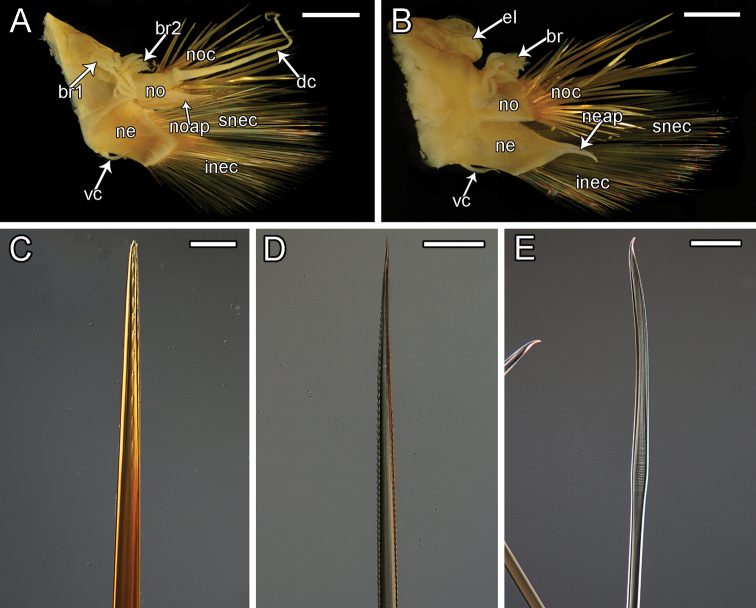
Micrographs of *P. goffrediae* sp. nov. holotype SIO-BIC A5485 **A** right parapodium from segment 10 **B** right parapodium from segment 11 **C** notochaeta **D** superior neurochaeta (supra-acicular) **E** inferior neurochaeta (subacicular). Abbreviations: br1, branchiae small group 1 attached to dorsal tubercle; br2, branchiae large group 2 attached near base of notopodium; noc, notochaetae; dc, dorsal cirrus; no, notopodium; snec, superior neurochaetae; ne, neuropodium; neap, neuroacicular process; noap, notoacicular process; vc, ventral cirrus; inec, inferior neurochaetae; el, elytrophore; br, single large group of branchiae on elytrigerous segment. Scale bars: 2 mm (**A, B**); 15 μm (**C, E**); 10 μm (**D**).

Figures 6H , 7C , 10C , 14 , 15

##### Type locality.

Whalefall in Monterey Canyon, California (36°36.79'N, 122°26.01'W), ROV “Tiburon” Dive 742, 2891 m depth, 29 September 2004.

##### Material examined.

**Type specimen: *Holotype*** (SIO-BIC A5485) from a whalefall in Monterey Canyon, California (36°36.79'N, 122°26.01'W), ROV “Tiburon” Dive 742, 2891 m depth, 29 September 2004; fixed in 95% ethanol and preserved in 50% ethanol, with a parapodium fixed and preserved in 95% ethanol. ***Paratype***: One specimen (SIO-BIC A5464) from the same location as holotype; fixed in formalin and preserved in 50% ethanol, with posterior segments 16–21 fixed and preserved in 95% ethanol.

##### Description.

In life, large, overlapping, iridescent light pink elytra covering the dorsum. Dorsum with ciliated transverse bands extending onto bases of elytrophores and dorsal tubercles. Chaetae extending beyond the width of elytra (Fig. 7C). Twenty-one segments total (Fig. 14A, B). Elytra and elytrophores large, bulbous, nine pairs, on segments 2, 4, 5, 7, 9, 11, 13, 15, 17 (Fig. 14A). Elytra sub-reniform, thick; greatly textured along the posterior margin, with several pointed macrotubercles (Figs 7C, 14D). Elytra on segments 2, 17 ca. 50–75% the size of mid-body elytra (Fig. 7C). Elytra on segment 17 curving to a lateral point in live specimen (Fig. 7C). Pharynx with seven dorsal border papillae and six ventral border papillae (Fig. 14C). Bilobed prostomium with triangular anterior lobes bearing short, thin, very delicate lateral antennae (= minute frontal filaments, sensu Pettibone 1993). Smooth median antenna with bulbous ceratophore in anterior notch. Eyes lacking. Pair of thick, smooth, tapering palps, ca. two and a half times the length of prostomium (Fig. 14E). Segment 1 with dorsal and ventral pairs of smooth, tapering anterior cirri (= tentacular cirri, sensu Pettibone 1993), ca. the same length as palps. Ventral anterior cirri slightly shorter than dorsal anterior cirri. Cirrophores of anterior cirri long and cylindrical, each with small acicular lobe on inner side (Fig. 14E, F). Smooth ventral cirri on segments 2–21. Buccal cirri of segment 2 modified, with bulbous ceratophores and longer styles, ca. three and a half times the length of remaining ventral cirri (Fig. 14F). Buccal cirri attached to base of neuropodia. Ventral cirri on segments 3–21 attached to middle of neuropodia, with bulbous ceratophores and short, tapering styles (Fig. 14B, F). Dorsal cirri present on non-elytrigerous segments 3, 6, 8, 10, 12, 14, 16, 18, 20, 21. Cirrophores of dorsal cirri cylindrical, rather long, fused to posterior sides of notopodia. Styles of dorsal cirri long, extending beyond length of chaetae. Segment 19 modified, lacking dorsal cirri and elytrophores (Fig. 14I). Arborescent branchiae compact, with relatively long terminal filaments, beginning on segment 2 (Fig. 14E) and continuing to segment 17 (Fig. 14I). Branchiae forming single large groups on elytrigerous segments, attached to bases of notopodia. Branchiae forming two groups on cirrigerous segments; small groups attached to dorsal tubercles and large groups attached near bases of notopodia (Fig. 14G). Three pairs of thin rounded folds of unknown function attached to anterior sides of neuropodia on segments 8–10. Four pairs of ventral segmental papillae on segments 12–15 (Fig. 14B). Ventral papillae rather long, slender, curved laterally, and followed by two pairs of lamellae (Fig. 14H). Rounded ventral lamellae have similar orientation as papillae but flattened and not protruding as much toward posterior end. Pygidium with a pair of anal cirri, long but not extending beyond the outline of the body (Fig. 14J). Parapodia biramous. Neuropodia ca. twice the length of notopodia, with an acicular process. On cirrigerous segments, notopodia with dorsal tubercles possessing small bundles of branchiae (Fig. 15A, B). Notopodia extending distally into acicular processes. Notochaetae forming radiating bundles, stout, with double rows of spines (Fig. 15C). Notochaetae almost as long as neurochaetae. Neurochaetae slender, forming fan-shaped bundles (Fig. 15A, B). Superior neurochaetae (supra-acicular) with double rows of spines (Fig. 15D). Inferior neurochaetae (sub-acicular) with double rows of teeth from the mid swelling to the hooked tips; smooth beneath the mid swelling (Fig. 15E). Inferior neurochaetae teeth are less prominent than the superior neurochaetae spines. Hooked jaws with small teeth on inner borders (Fig. 10C).

##### Morphological variation.

Holotype is 39 mm long, 27 mm wide, including chaetae. Paratype is 43 mm long (segments 1–15), 26 mm wide, including chaetae.

##### Remarks.

*Peinaleopolynoe goffrediae* sp. nov.’s closest relative is *P. orphanae* sp. nov. (Fig. 1). *Peinaleopolynoe goffrediae* sp. nov. can be distinguished from *P. orphanae* sp. nov. by the segmental range of branchiae, the former present on segments 2–17 and the latter on segments 3–18 (Table 5). Additionally, the four pairs of ventral papillae on *P. goffrediae* sp. nov. are long, tapered, and curved laterally, distinguishing it from the small, rounded, cylindrical papillae of *P. orphanae* sp. nov. *Peinaleopolynoe goffrediae* sp. nov. is unique among *Peinaleopolynoe* taxa in that the angle formed by the ventral part on the neuroacicular lobe is clearly diagonal while it is nearly horizontal in the other species.

##### Etymology.

*Peinaleopolynoe goffrediae* sp. nov. is named after Dr. Shana K. Goffredi for her notable contribution to the exploration and research of deep-sea chemosynthetic ecosystems (especially whalefalls), focusing on symbiotic relationships between bacteria and marine invertebrates.

##### Ecology.

*Peinaleopolynoe goffrediae* sp. nov. was only found associated with a whalefall (Table 5). Fig. 6H shows the holotype observed *in situ* on a whale carcass before collection.

#### Peinaleopolynoe mineoi

Taxon classificationAnimaliaPhyllodocida

Hatch & Rouse sp. nov.

118D4417-5A61-57D4-88E5-B3551A7AF18C

http://zoobank.org/2D5CFC88-113C-4DBC-8EC1-72D5641FFB04

**Figure 16.**
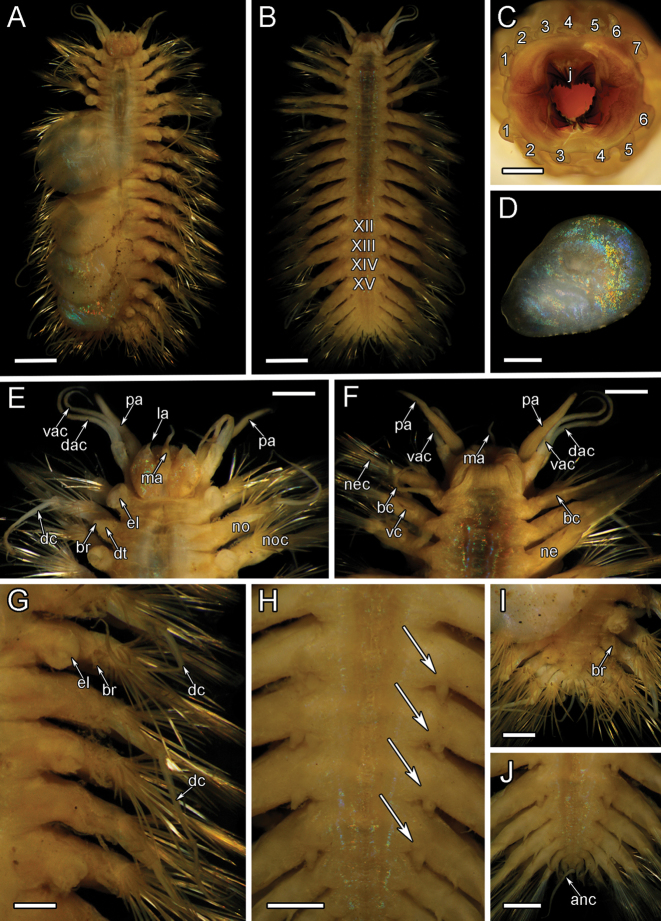
Micrographs of *P. mineoi* sp. nov. holotype SIO-BIC A10071 and paratype SIO-BIC A9709 **A** dorsal view, holotype **B** ventral view, holotype. Segments 12–15 are marked to indicate the presence of four pairs of papillae **C** frontal view of proboscis showing papillae and jaws, paratype. Numbers mark the papillae on the dorsal (seven papillae) and ventral (six papillae) surfaces **D** loose elytron, holotype **E** dorsal view of anterior, holotype **F** ventral view of anterior, holotype **G** right side branchiae on segments 8–13, holotype **H** ventral papillae on segments 12–15 (four pairs) indicated by arrows, holotype **I** dorsal view of posterior, holotype **J** ventral view of posterior, holotype. Abbreviations: XII, segment 12; XIII, segment 13; XIV, segment 14; XV, segment 15; j, jaws; ma, median antenna; la, lateral antenna; pa, palp; dac, dorsal anterior cirrus; vac, ventral anterior cirrus; el, elytrophore; br, branchiae; nec, neurochaetae; noc, notochaetae; dc, dorsal cirrus; no, notopodium; vc, ventral cirrus; bc, buccal cirrus; ne, neuropodium; dt, dorsal tubercle; anc, anal cirrus. Scale bars: 1 mm (**A, B, D**); 0.5 mm (**C, E–J**).

**Figure 17.**
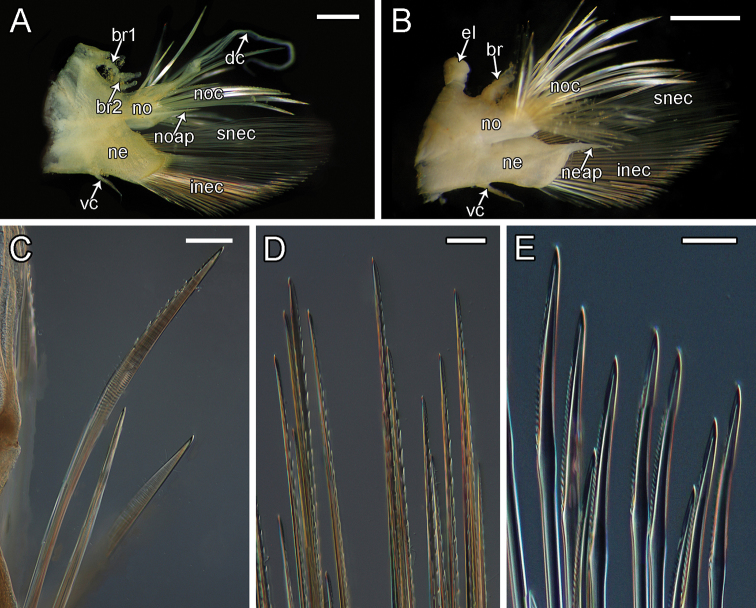
Micrographs of *P. mineoi* sp. nov. holotype SIO-BIC A10071 **A** right parapodium from segment 10 **B** right parapodium from segment 11 **C** notochaetae **D** superior neurochaetae (supra-acicular) **E** inferior neurochaetae (subacicular). Abbreviations: br1, branchiae small group 1 attached to dorsal tubercle; br2, branchiae large group 2 attached near base of notopodium; noc, notochaetae; dc, dorsal cirrus; no, notopodium; snec, superior neurochaetae; ne, neuropodium; neap, neuroacicular process; noap, notoacicular process; vc, ventral cirrus; inec, inferior neurochaetae; el, elytrophore; br, single large group of branchiae on elytrigerous segment. Scale bars: 0.5 mm (**A, B**); 15 μm (**C**); 5 μm (**D, E**).

Figures 7D , 10D , 16 , 17

##### Type locality.

Mound 12, Costa Rica (8°55.99'N, 84°18.45'W), ROV “SuBastian” Dive S0215, 1011 m depth, 8 January 2019.

##### Material examined.

**Type specimen: *Holotype*** (SIO-BIC A10071) on bones deployed at Mound 12, Costa Rica (8°55.99'N, 84°18.45'W), ROV “SuBastian” Dive S0215, 1011 m depth, 8 January 2019; fixed and preserved in 95% ethanol. ***Paratypes***: One specimen (SIO-BIC A10070) from the same location as holotype; fixed and preserved in 95% ethanol. One specimen (SIO-BIC A9709) from on bones deployed at Mound 12, Costa Rica (8°55.8'N, 84°18.70'W), HOV “Alvin” Dive AD4974, 992 m depth, 20 October 2018; fixed and preserved in 95% ethanol. One specimen (MZUCR 1001-01) (SIO-BIC A9919) on a piece of decayed wood found at Mound 11, Costa Rica (8°55.33'N, 84°18.27'W), HOV “Alvin” Dive AD4988, 1010 m depth, 3 November 2018; fixed in formalin and preserved in 50% ethanol, with elytra fixed and preserved in 95% ethanol.

##### Description.

In life, large, overlapping, iridescent, semi-transparent elytra covering the dorsum. Dorsum with ciliated transverse bands extending onto bases of elytrophores and dorsal tubercles. Chaetae extending beyond the width of elytra (Fig. 7D). Twenty-one segments total (Fig. 16A, B). Elytra and elytrophores large, bulbous, nine pairs, on segments 2, 4, 5, 7, 9, 11, 13, 15, 17 (Fig. 16A). Elytra sub-reniform, very thin, with several rounded broad macrotubercles along the posterior margin (Figs 7D, 16D). Pharynx with seven dorsal border papillae and six ventral border papillae (Fig. 16C). Bilobed prostomium with triangular anterior lobes bearing short, thin, very delicate lateral antennae (= minute frontal filaments, sensu Pettibone 1993). Smooth median antenna with bulbous ceratophore in anterior notch. Eyes lacking. Pair of thick, smooth, tapering palps (Fig. 16E). Segment 1 with dorsal and ventral pairs of smooth, tapering anterior cirri (= tentacular cirri, sensu Pettibone 1993), approximately the same length as palps. Ventral anterior cirri slightly shorter than dorsal anterior cirri. Cirrophores of anterior cirri long and cylindrical, each with small acicular lobe on inner side (Fig. 16E, F). Smooth ventral cirri on segments 2–21. Buccal cirri of segment 2 modified, with bulbous ceratophores and longer styles, ca. four times the length of remaining ventral cirri (Fig. 16F). Buccal cirri attached to base of neuropodia. Ventral cirri on segments 3–21 attached to middle of neuropodia, with bulbous ceratophores and short, tapering styles (Fig. 16B, F). Dorsal cirri present on non-elytrigerous segments 3, 6, 8, 10, 12, 14, 16, 18, 20, 21. Cirrophores of dorsal cirri cylindrical, rather long, fused to posterior sides of notopodia. Styles of dorsal cirri long, extending beyond length of chaetae. Segment 19 modified, lacking dorsal cirri and elytrophores (Fig. 16I). Arborescent branchiae small with thin terminal filaments, beginning on segment 3 (Fig. 16E) and continuing to segment 16 (Fig. 16I). Branchiae forming single groups on elytrigerous segments, attached to bases of notopodia. Branchiae forming two groups on cirrigerous segments; small groups attached to dorsal tubercles and larger groups attached near bases of notopodia (Fig. 16G). Four pairs of ventral segmental papillae on segments 12–15 (Fig. 16B); medium length, curved laterally (Fig. 16H). Pygidium with a pair of relatively short anal cirri (Fig. 16J). Parapodia biramous. Neuropodia ca. twice the length of notopodia, with an acicular process. On cirrigerous segments, notopodia with dorsal tubercles possessing small bundles of branchiae (Fig. 17A, B). Notopodia extending distally into acicular processes. Notochaetae forming radiating bundles, stout, with double rows of spines (Fig. 17C). Notochaetae almost as long as neurochaetae. Neurochaetae slender, forming fan-shaped bundles (Fig. 17A, B). Superior neurochaetae (supra-acicular) with double rows of spines and very slightly curved tips (Fig. 17D). Inferior neurochaetae (sub-acicular) with double rows of teeth from the mid swelling to the hooked tips; smooth beneath the mid swelling (Fig. 17E). Inferior neurochaetae teeth are less prominent than the superior neurochaetae spines. Hooked jaws with large, rounded, protruding teeth on inner borders (Fig. 10D).

##### Morphological variation.

Holotype is 14 mm long, 7 mm wide, including chaetae. Paratypes range from 13–15 mm long, 5–7 mm wide, including chaetae.

##### Remarks.

*Peinaleopolynoe mineoi* sp. nov. is the sister taxon to the remaining *Peinaleopolynoe* spp. (Fig. 1) and like most of them has nine pairs of elytra and segment 19 lacking dorsal cirri and elytrophores (Table 5). Additionally, the four pairs of ventral papillae are tapering and curved laterally, as in all *Peinaleopolynoe* spp., excluding *P. orphanae* sp. nov. The angle of the ventral part of the neuroacicular lobe is nearly horizontal, distinct from *P. goffrediae* sp. nov. (Table 5). *Peinaleopolynoe mineoi* sp. nov. possesses several broadly rounded macrotubercles on the posterior margin of the elytra, quite distinct from the pointed macrotubercles found in *P. santacatalina* and *P. goffrediae* sp. nov., and the single, pointed macrotubercle found in *P. elvisi* sp. nov. (Table 5). *Peinaleopolynoe mineoi* sp. nov. is unique among *Peinaleopolynoe* taxa in having large, rounded, protruding teeth on the inner borders of their jaws.

##### Etymology.

*Peinaleopolynoe mineoi* sp. nov. is named after Ronald M. Mineo, MD, in recognition of support from the Mineo family, their interest in the deep sea, and support for our research.

##### Ecology.

*Peinaleopolynoe mineoi* sp. nov. was found associated with bones and wood (Table 5). Like *P. santacatalina* and *P. orphanae* sp. nov., it may be more of a habitat generalist than other *Peinaleopolynoe*.

## Discussion

In this study we provided new data for five loci (16S, CytB, 18S, 28S and H3) for a series of specimens that had previously been documented for only COI in Goffredi et al. (2017). That study was a biodiversity inventory of vents in the southern Gulf of California and included COI data for previously described polynoids such as *B. hessleri* (the type species for genus), *B. sandersi*, *B. cupreus* (a monotypic genus), *L. fimbriatum* (the type species for genus), *Lepidonotopodium williamsae* Pettibone, 1984, an undescribed *Lepidonotopodium* sp., and a previously undescribed *Peinaleopolynoe* (here described as *P. orphanae* sp. nov.). We also have added here a new 16S sequence for *B. guaymasensis* to supplement the previous 18S and COI data from Glover et al. (2005). Seven COI sequences were generated for *B. sandersi* from the type locality (Galápagos) and these matched the *B. sandersi* data from the Gulf of California, confirming the identification in Goffredi et al. (2017). We also provided the first DNA data for the only branchiate genus for which such data was lacking, *T. branchiata*. This new data, combined with the new data for *Peinaleopolynoe* spp. allowed for a further assessment of the relationship among deep-sea polynoids associated with vents, seeps and food falls (see below).

The prime focus of this study was *Peinaleopolynoe* and we generated DNA data for the two described species *P. sillardi* and *P. santacatalina*, and a series of undescribed species in addition to the *Peinaleopolynoe* reported in Goffredi et al. (2017). The monophyly of *Peinaleopolynoe* was supported here by the phylogenetic analysis of DNA data (Fig. 1), as well as by the presence of ventral papillae on segments 12–15 (Table 5, Fig. 4A). In addition to the phylogenetic results, species delimitation is supported by morphology (Table 5) and the marked difference in the uncorrected COI distance analysis; the intraspecific COI distances range from 0–1.46%, while the interspecific COI distances range from 12.65–19.64% (Table 4). These distances are in excess of what has often been used to delineate species level taxa in annelids (see review by Nygren 2014). We also were able to find apomorphic features for all four new species (Table 5): the branchiae of *P. orphanae* sp. nov. terminate on segment 18 and the four pairs of ventral papillae are small, cylindrical, and rounded; *P. elvisi* sp. nov. has six pairs of border papillae on the pharynx and a single macrotubercle on the elytra; the ventral part of the neuroacicular lobe is diagonal in *P. goffrediae* sp. nov.; and *P. mineoi* sp. nov. has large, rounded, protruding teeth on the inner borders of their jaws. The discovery of these four new species takes the number of *Peinaleopolynoe* spp. to six. The *Peinaleopolynoe* clade was recovered as sister group (Fig. 1) to what we refer to here as *Branchinotogluma* clade 5 (*B. bipapillata* and *B.* sp. nov. 1). Although the state of elytral number for the common ancestor between *Peinaleopolynoe* and *Branchinotogluma* clade 5 was unresolved (Fig. 4B), we conclude that it is likely ten pairs of elytra. The sister group to the combined *Branchinotogluma* clade 5 with *Peinaleopolynoe* is *B. japonicus* with *Branchipolynoe* (Fig. 1), which all possess ten pairs of elytra (Miura and Hashimoto 1991; Zhang et al. 2018a; Lindgren et al. 2019). The most likely ancestral state for *Peinaleopolynoe* is nine pairs of elytra, supporting a reversal in *P. santacatalina*, which is the only *Peinaleopolynoe* species that possesses ten elytra in the clade (Fig. 4B).

The first two *Peinaleopolynoe* spp., *P. sillardi* and *P. santacatalina*, were described from organic falls, and Desbruyères and Laubier (1988) highlighted this preference in the genus name, which includes a reference to hunger or being famished. This preferred habitat is indeed unusual among the deep-sea polynoids to which *Peinaleopolynoe* is closely related, where hydrothermal vents and methane seeps are the normal habitat. An exception is *B. guaymasensis*, which is known from whalefalls and hydrothermal vents (Pettibone 1989; Glover et al. 2005). Table 5 summarizes the habitats for the six *Peinaleopolynoe* now known and it is notable that with the exception of *P. orphanae* sp. nov., all of these ‘hungry’ scale worms have been found on organic remains such as whalefalls, deployed bones and wood. Unusually, *P. santacatalina* was also found at a seep, and the derived position of *P. orphanae* sp. nov. within *Peinaleopolynoe* suggests its occurrence at seeps and vents may be a secondary colonization.

Our addition of DNA data for new taxa and additional loci for previously published specimens (Table 1) has allowed for an updated assessment of the phylogeny of the clade of deep-sea polynoids that are mainly found at vents, seeps, and organic falls. Our results (Fig. 1, Suppl. material 1: Figs S1, S2) are largely similar to the three gene phylogeny in Zhou et al. (2018: fig. 7), though our rooting is different. We show that *B. cupreus*, which has branchiae, is the sister group to all of the other ingroup taxa. Pettibone (1985b) had recognized that the branchiae of *B. cupreus* were quite different from those of the only known branchiate polynoid at that time, *Branchipolynoe*, and placed it in its own subfamily Branchiplicatinae. We found *Branchinotogluma* to be paraphyletic, as has been reported by others recently (Zhang et al. 2018a, b; Zhou et al. 2018; Wu et al. 2019). *Branchinotogluma* occurs in seven places across our phylogeny (Fig. 1). The type species of *Branchinotogluma* is *B. hessleri*, which occupies an isolated position in Fig. 1. If this position is maintained with further phylogenetic investigation, then membership of *Branchinotogluma* may become quite restricted. Support for some keys nodes is low though (Fig. 1), so no taxonomic changes are recommended at this time for *Branchinotogluma*.

A clade of mainly non-branchiate polynoids *Levensteiniella* spp., *Lepidonotopodium* spp., *B. guaymasensis* and *T. branchiata* (Fig. 1) was sister group to *B. segonzaci* and nested within a clade of various branchiate polynoids, suggesting they may have lost this feature. *Thermopolynoe branchiata* does have branchiae, but in his description Miura (1994) pointed out that this species was unique among branchiate polynoids in having well-developed bracts encircling the notopodia and in the position of branchiae. *Thermopolynoe branchiata* has arborescent branchiae as in *Branchinotogluma*, *Branchipolynoe* and *Peinaleopolynoe*, but segments with two groups of branchiae are split into anterior and posterior groups as opposed to upper and lower groups (Miura 1994). It is thus clear that branchiae have evolved and been lost several times in Polynoidae. The placement of *L. fimbriatum* as sister taxon to *T. branchiata* and the other three included *Lepidonotopodium* terminals as sister group to *Levensteiniella* suggest *Lepidonotopodium* will require revision. Further taxon sampling of the other species in *Lepidonotopodium* is warranted and support for some key nodes is too low at present.

In a recent phylogenetic study of deep-sea Polynoidae, Bonifácio and Menot (2018) made the polynoid subfamilies that comprise the majority of deep-sea polynoids found at vents, seeps and whalefalls, namely Branchinotogluminae, Branchiplicatinae, Branchipolynoinae, and Lepidonotopodinae, junior synonyms of Macellicephalinae. The sampling of these taxa for a morphology/molecular sequence data analysis (Bonifácio and Menot 2018: fig. 2) that they had for these subfamilies (*L. fimbriatum*, *B. sandersi*, *B. symmytilida* and *Peinaleopolynoe* sp.) resulted in a clade referred to as ‘clade b1’ that formed the sister group to the rest of the Macellicephalinae. Clade b1 also included *B. guaymasensis* that was placed in Macellicephalinae when first described by Pettibone (1989). Our results shown here clearly suggest that the members of Branchinotogluminae, Branchiplicatinae, Branchipolynoinae, and Lepidonotopodinae, as well as *B. guaymasensis* and *Levensteiniella* (initially described as Macellicephalinae), form a well-supported clade. Here, we refer to this clade, clade b1 of Bonifácio and Menot (2018), with the oldest available subfamily name and reinstate Lepidonotopodinae.

## Supplementary Material

XML Treatment for Lepidonotopodinae

XML Treatment for Peinaleopolynoe

XML Treatment for Peinaleopolynoe santacatalina

XML Treatment for Peinaleopolynoe orphanae

XML Treatment for Peinaleopolynoe elvisi

XML Treatment for Peinaleopolynoe goffrediae

XML Treatment for Peinaleopolynoe mineoi

## Supplementary materials

Supplementary material 1 Figures S1, S2 Explanation note **Figure S1.** Bayesian inference (BI) tree of the combined analysis from six genes (COI, 16S, 18S, 28S, H3, CytB) aligned with MAFFT and then concatenated. Numbers next to nodes are BI posterior probability. Key: * indicates 0.95 posterior probability or greater. **Figure S2.** Strict consensus tree of the two most parsimonious trees using the combined analysis from six genes (COI, 16S, 18S, 28S, H3, CytB) aligned with MAFFT and then concatenated. Numbers next to nodes are MP jackknife support values. Key: * indicates 95% jackknife or greater. https://binary.pensoft.net/file/409288This dataset is made available under the Open Database License (http://opendatacommons.org/licenses/odbl/1.0/). The Open Database License (ODbL) is a license agreement intended to allow users to freely share, modify, and use this Dataset while maintaining this same freedom for others, provided that the original source and author(s) are credited.Avery S. Hatch, Haebin Liew, ﻿Stéphane Hourdez, Greg W. Rouse

Supplementary material 2 In situ fighting behavior of *Peinaleopolynoe orphanae* sp. nov. observed in the Pescadero Basin, Gulf of California, Mexico Data type movie https://binary.pensoft.net/file/409289This dataset is made available under the Open Database License (http://opendatacommons.org/licenses/odbl/1.0/). The Open Database License (ODbL) is a license agreement intended to allow users to freely share, modify, and use this Dataset while maintaining this same freedom for others, provided that the original source and author(s) are credited.Avery S. Hatch, Haebin Liew, ﻿Stéphane Hourdez, Greg W. Rouse
